# Predicting alcohol use disorder risk in firefighters using a multimodal deep learning model: a cross-sectional study

**DOI:** 10.3389/fpsyt.2025.1643552

**Published:** 2025-11-03

**Authors:** MyeongGyun Jang, DongOk Kim, Sujung Yoon, Hwamin Lee

**Affiliations:** ^1^ Department of Biomedical Informatics, Korea University College of Medicine, Seoul, Republic of Korea; ^2^ Ewha Brain Institute, Ewha Womans University, Seoul, Republic of Korea; ^3^ Department of Brain and Cognitive Sciences, Ewha Womans University, Seoul, Republic of Korea

**Keywords:** alcohol use disorder, firefighters, multimodal deep learning, structural MRI, occupational psychiatry, neuroimaging biomarkers

## Abstract

**Introduction:**

Firefighters constitute a high-risk occupational cohort for alcohol use disorder (AUD) due to chronic trauma exposure, yet traditional screening methodologies relying on self-report instruments remain compromised by systematic underreporting attributable to occupational stigma and career preservation concerns. This cross-sectional investigation developed and validated a multimodal deep learning framework integrating T1-weighted structural magnetic resonance imaging with standardized neuropsychological assessments to enable objective AUD risk stratification without necessitating computationally intensive functional neuroimaging protocols.

**Methods:**

Analysis of 689 active-duty firefighters (mean age 43.3±8.8 years; 93% male) from a nationwide occupational cohort incorporated high-resolution three-dimensional T1-weighted structural MRI acquisition alongside comprehensive neuropsychological evaluation utilizing the Grooved Pegboard Test for visual-motor coordination assessment and Trail Making Test for executive function quantification. The novel computational architecture synergistically combined ResNet-50 convolutional neural networks for hierarchical morphological feature extraction, Vision Transformer modules for global neuroanatomical pattern recognition, and multilayer perceptron integration of clinical variables, with model interpretability assessed through Gradient-weighted Class Activation Mapping and SHapley Additive exPlanations methodologies. Performance evaluation employed stratified three-fold cross-validation with DeLong's test for statistical comparison of receiver operating characteristic curves.

**Results:**

The multimodal framework achieved 79.88% classification accuracy with area under the receiver operating characteristic curve of 79.65%, representing statistically significant performance enhancement relative to clinical-only (62.53%; p<0.001) and neuroimaging-only (61.53%; p<0.001) models, demonstrating a 17.35 percentage-point improvement attributable to synergistic cross-modal integration rather than simple feature concatenation. Interpretability analyses revealed stochastic activation patterns in unimodal neuroimaging models lacking neuroanatomically coherent feature localization, while clinical feature importance hierarchically prioritized biological sex and motor coordination metrics as primary predictive indicators. The framework maintained robust calibration across probability thresholds, supporting operational feasibility for clinical deployment.

**Discussion:**

This investigation establishes that structural neuroimaging combined with targeted neuropsychological assessment achieves classification performance comparable to complex multimodal protocols while substantially reducing acquisition time and computational requirements, offering a pragmatic pathway for implementing objective AUD screening in high-risk occupational populations with broader implications for psychiatric risk stratification in trauma-exposed professions.

## 1 Introduction

Firefighters constitute a distinct occupational group regularly exposed to life-threatening emergencies and cumulative psychological trauma including fire suppression, technical rescues, hazardous material responses, and mass casualty incidents. This continuous exposure imposes substantial psychological and physiological burdens, placing firefighters at elevated risk for a range of mental health disorders, most notably alcohol use disorder (AUD) (1). Epidemiological studies have consistently reported higher rates of problematic alcohol consumption among firefighters compared to the general population, a disparity that persists even after adjusting for demographic and socioeconomic factors (2–4).

Beyond alcohol-specific outcomes, large-scale evidence from Canadian public safety personnel (PSP) shows substantially elevated screening rates for common mental disorders relative to the general population. In a national survey of 5,813 PSP, Carleton et al. (5) reported that 15.1% screened positive for at least one current disorder and 26.7% for two or more, with meaningful differences across PSP categories (5). These findings underscore the high and heterogeneous mental health burden in firefighters’ broader occupational context and help explain why coping-motivated alcohol use often emerges in this workforce, reinforcing the need for objective, stigma-resistant risk assessment beyond self-report. This pattern is consistent with evidence that public safety personnel, including firefighters, frequently engage in coping-motivated alcohol use in response to trauma and chronic operational stress (6–9), further strengthening the rationale for objective risk assessment methods. The etiology of AUD within this population is multifaceted, reflecting interactions among neurobiological predispositions, occupational stress, and psychosocial dynamics. Alcohol is often utilized as a maladaptive coping strategy to manage symptoms of hyperarousal, intrusive memories, and emotional distress stemming from repeated trauma exposure (7–9). Over time, this reliance on alcohol for emotional regulation can lead to reinforcement cycles that escalate into habitual and dependent use (10). These clinical risk pathways are further compounded by occupational culture. Firefighting environments frequently normalize post-shift drinking and valorize stoicism, creating a paradox in which alcohol use is both institutionally sanctioned and individually stigmatized (2, 10). Consequently, many firefighters refrain from help-seeking behaviors and underreport their alcohol consumption due to fears of career-related repercussions.

The implications of AUD within firefighting populations extend beyond individual health, impacting operational readiness, decision-making under pressure, and public safety during emergency response. Excessive alcohol use among first responders in high-stakes environments is linked to increased risk-taking behaviors, such as driving while intoxicated, thereby contributing to significant occupational problems that can affect team performance, and posing severe threats to personal safety, including heightened suicidality and increased risk of traumatic incidents (11). Despite these risks, early detection of alcohol misuse remains challenging. Current screening protocols rely heavily on self-reported questionnaires such as the Alcohol Use Disorder Identification Test (AUDIT), which are vulnerable to social desirability bias, impression management, and concerns regarding occupational repercussions (12). Furthermore, cultural norms emphasizing resilience and self-reliance may suppress disclosure of substance use and deter engagement with support services (13). Accordingly, there is a clear need for objective, stigma-resistant screening approaches that integrate biological and behavioral indicators rather than relying solely on self-report.

Recent advances in neuroimaging and machine learning have opened new avenues for objective assessment of psychiatric disorders. Structural MRI markers have been shown to correlate with various psychiatric phenotypes, including those related to substance use disorders (14). Machine learning techniques applied to neuroimaging data have demonstrated promising diagnostic and predictive accuracy across various psychiatric disorders (15, 16). However, their application to alcohol use risk prediction within occupational cohorts remains underexplored. Within high-risk occupational cohorts such as firefighters, studies that objectively predict AUD risk by integrating structural MRI with standardized neuropsychological measures remain scarce.

To address this gap, the present study proposes a multimodal deep learning approach that integrates neuroimaging features with clinical and cognitive measures to predict AUD risk in a national sample of active-duty firefighters. This method aims to overcome limitations of conventional self-report tools by leveraging biologically informed, data-driven markers to enhance early identification of high-risk individuals. Through this integration, we seek to contribute to the development of precision screening strategies tailored to the unique demands and vulnerabilities of high-stress emergency response professionals.

## 2 Materials and methods

### 2.1 Study design and participants

This study utilized a cross-sectional design to develop and evaluate a multimodal deep learning framework for predicting alcohol use disorder (AUD) risk in an occupational cohort of active-duty firefighters in the Republic of Korea. Participants were recruited from multiple fire stations nationwide. Eligibility criteria included: age 25–65 years, active employment as a firefighter, and availability of both T1-weighted structural magnetic resonance imaging (MRI) and complete clinical assessment data. Exclusion criteria comprised a history of neurological disorders (e.g., epilepsy, stroke, traumatic brain injury), major psychiatric conditions other than AUD, current use of psychotropic medications, MRI-detected structural brain abnormalities, or contraindications to MRI scanning (e.g., metallic implants, claustrophobia).

**Figure 1:**
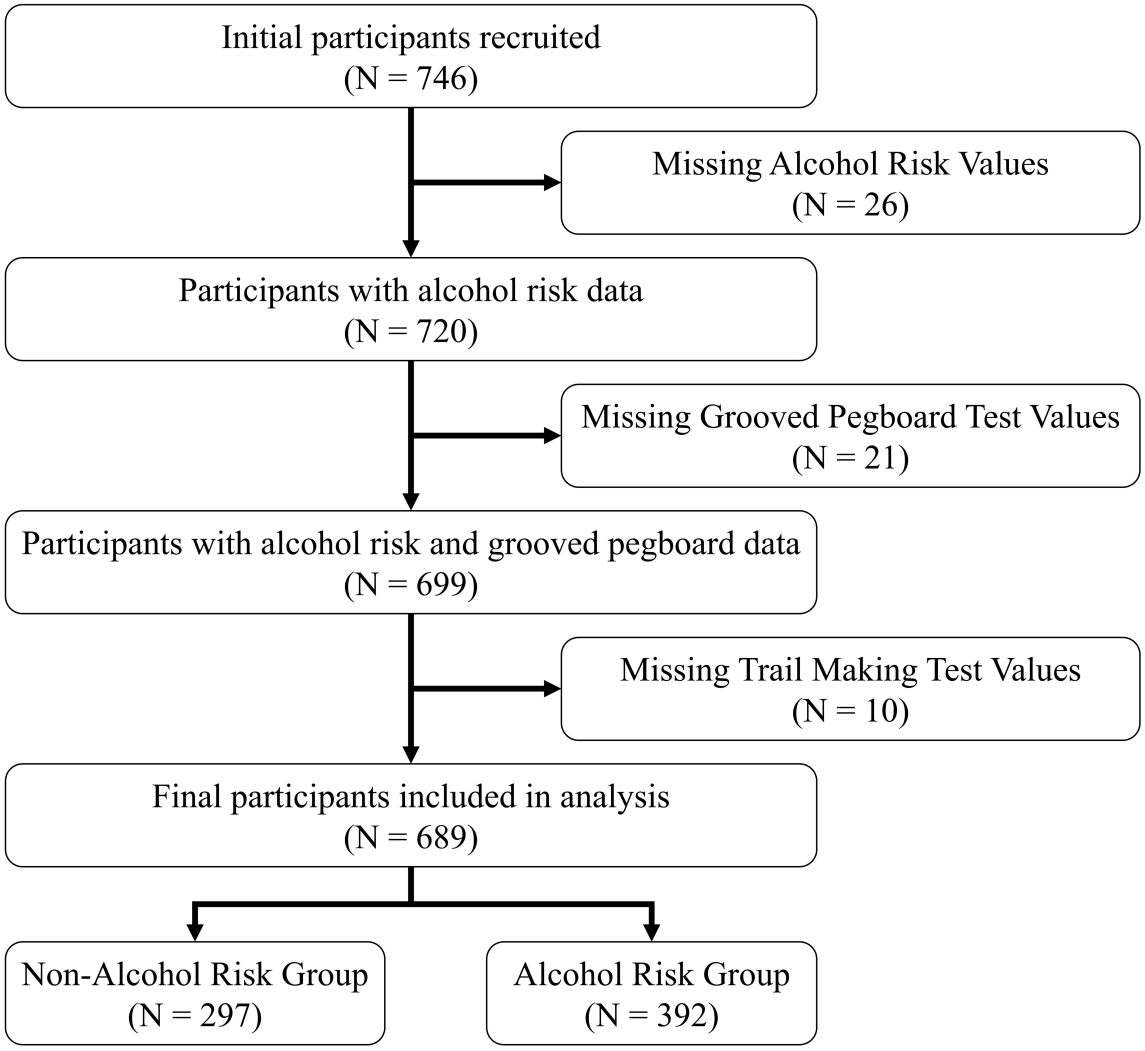
Participant recruitment and data preprocessing workflow for the firefighter cohort.

Of 746 initially enrolled firefighters, 35 were excluded due to incomplete imaging data, 14 for missing clinical assessments, and 8 for MRI-detected structural anomalies, resulting in a final analytical sample of 689 participants (mean age 43.3 ± 8.8 years; 93% male). **Figure 1** illustrates the participant recruitment and data preprocessing workflow. All participants provided written informed consent, and the study protocol was approved by the Institutional Review Board of Ewha Womans University. The research adhered to the ethical principles of the Declaration of Helsinki.

### 2.2 Clinical assessments

Cognitive and motor functions were evaluated using two standardized neuropsychological tests: the Grooved Pegboard Test and the Trail Making Test (TMT) (17, 18) (19, 20). The Grooved Pegboard Test assessed visual-motor coordination and fine motor control. Participants inserted 25 uniquely shaped pins into corresponding grooves as quickly as possible, with completion times (seconds) recorded for both dominant and non-dominant hands; longer times indicated poorer performance (17, 18). The TMT evaluated processing speed, cognitive flexibility, and executive function. Part A required participants to connect numbered circles sequentially, while Part B involved alternating between numbers and letters in ascending order. Completion times were recorded, with higher values reflecting lower cognitive efficiency (19, 20). Tests were administered by trained personnel under standardized conditions.

Alcohol use risk was assessed using the Alcohol Use Disorder Identification Test (AUDIT), a 10-item self-report questionnaire developed by the World Health Organization to evaluate alcohol consumption, dependence symptoms, and related harm (21). Scores range from 0 to 40, with a cutoff of ≥8 indicating hazardous drinking risk (22). The AUDIT was completed under supervised conditions to ensure data integrity.

### 2.3 Demographic and neuropsychological characteristics

To align comparisons with standard occupational screening practice, we stratified the cohort using the established AUDIT cut-off (≥8 vs<8). **Table 1** summarizes the demographic and neuropsychological characteristics of the study cohort, stratified by AUDIT-based alcohol risk status (≥8: alcohol risk, n=392, 56.9%;<8: non-alcohol risk, n=297, 43.1%). The alcohol risk group had a mean age of 43.16 ± 8.53 years, compared to 42.58 ± 8.67 years for the non-alcohol risk group, with no significant difference (p=0.380, two-tailed independent samples t-test). We used two-tailed independent-samples t-tests for continuous variables because the groups are non-overlapping at the participant level, the t-test provides an efficient test of mean differences, and with our sample size it is reasonably robust to moderate deviations from normality; a two-sided test also guards against effects in either direction. A significant gender disparity was observed (p<0.001, two-tailed Pearson Chi-square test), with the alcohol risk group showing higher male predominance (380 males, 12 females) compared to the non-alcohol risk group (257 males, 40 females). The Pearson chi-square test was chosen for categorical comparisons (e.g., sex distribution) because it assesses association between group membership and categorical outcomes without requiring distributional assumptions beyond adequate expected cell counts.

**Table 1:** Participant characteristics stratified by alcohol use risk status.

Variables	Alcohol risk	Non-alcohol risk	p value
Sample size	392	297	
Age (years) ^a^	43.16 ± 8.53	42.58 ± 8.67	0.380 ^b^
Gender (male/female)	380/12	257/40	< 0.001 ^c^
Grooved pegboard test			
Dominant ^a^	66.27 ± 8.45	67.27 ± 9.46	0.153 ^b^
Non-dominant ^a^	72.04 ± 9.49	72.48 ± 9.79	0.546 ^b^
Trail making test			
Test A ^a^	29.65 ± 7.78	28.71 ± 7.50	0.110 ^b^
Test B ^a^	74.69 ± 27.28	73.42 ± 25.25	0.527 ^b^

a Data are presented as mean ± standard deviation.

b *p* by two-tailed independent samples t-test.

c *p* by two-tailed Pearson Chi-square test.

Neuropsychological performance was comparable between groups. For the Grooved Pegboard Test, dominant hand completion times were 66.27 ± 8.45 seconds (alcohol risk) versus 67.27 ± 9.46 seconds (non-alcohol risk; p=0.153), and non-dominant hand times were 72.04 ± 9.49 seconds versus 72.48 ± 9.79 seconds (p=0.546). For the TMT, Part A completion times were 29.65 ± 7.78 seconds (alcohol risk) versus 28.71 ± 7.50 seconds (non-alcohol risk; p=0.110), and Part B times were 74.69 ± 27.28 seconds versus 73.42 ± 25.25 seconds (p=0.527). Interpreted under these method choices, the absence of significant between-group differences suggests that AUD risk, as defined by screening criteria, may precede measurable neuropsychological deficits in this occupational cohort.

### 2.4 MRI acquisition

Structural brain MRI scans were acquired using a 3.0 Tesla Philips MRI system (Philips Healthcare, Best, The Netherlands) equipped with a 32-channel head coil. High-resolution three-dimensional T1-weighted images were obtained with the following parameters: repetition time (TR) = 7.4 ms, echo time (TE) = 3.4 ms, flip angle = 8°, voxel size = 1 × 1 × 1 mm³, and 180 sagittal slices. All participants were instructed to maintain stillness and neutral head positioning throughout the scanning session to ensure image quality.

### 2.5 Data preprocessing

T1-weighted MRI data were preprocessed using the FMRIB Software Library (FSL, version 6.0 (23) to ensure standardized spatial normalization and artifact minimization. The preprocessing pipeline included both linear and nonlinear registration of each participant’s structural MRI to the Montreal Neurological Institute (MNI152) standard space, followed by resampling to a voxel resolution of 2 × 2 × 2 mm³. Following normalization, skull stripping was performed using the High-Definition Brain Extraction Tool (HD-BET), a deep learning–based algorithm designed to enhance the accuracy of brain tissue isolation from non-brain elements (24). This process improved visualization of key anatomical regions, including gray matter, white matter, and ventricular structures, while simultaneously reducing noise and enhancing segmentation fidelity. After skull stripping, the three-dimensional MRI volumes were segmented into 80 two-dimensional axial slices per participant, ensuring standardized anatomical coverage. The use of axial slices provides distinct spatial perspectives and clinically relevant information, facilitating detailed neuroanatomical interpretation and enabling precise detection of structural abnormalities or pathologies (25, 26).

To increase model generalizability, data augmentation techniques were applied. RandomAffine transformations introduced rotational variations (± 10°) and translation shifts (± 5%). ColorJitter transformations adjusted image brightness and contrast (± 20%), and RandomRotation transformations applied further rotational variation (± 15°) to simulate clinical variability. Pixel intensity normalization was performed using standardized mean and standard deviation values derived from large-scale neuroimaging datasets to standardize input distributions prior to model training (27–29).

Clinical assessment data underwent systematic preprocessing to ensure data integrity and model compatibility. Missing value analysis was conducted across all clinical variables, including demographic parameters (age, sex), neuropsychological test scores (Grooved Pegboard Test completion times for dominant and non-dominant hands, Trail Making Test Parts A and B), and alcohol use risk indicators (AUDIT scores). Participants with incomplete clinical assessments were excluded from the analytical cohort through listwise deletion, maintaining the methodological rigor requisite for multimodal integration. This conservative approach to missing data management, while potentially reducing statistical power, preserved the validity of cross-modal feature relationships critical to the multimodal learning framework. No imputation strategies were employed to avoid introducing artificial correlations between neuroimaging and clinical features. All continuous clinical variables were retained in their original scales to preserve interpretability, with normalization performed internally within the deep learning architecture through batch normalization layers. Categorical variables, specifically biological sex, were encoded using binary representation (0 = male, 1 = female) consistent with standard practices in medical machine learning applications.

### 2.6 Multimodal deep learning framework

**Figure 2:**
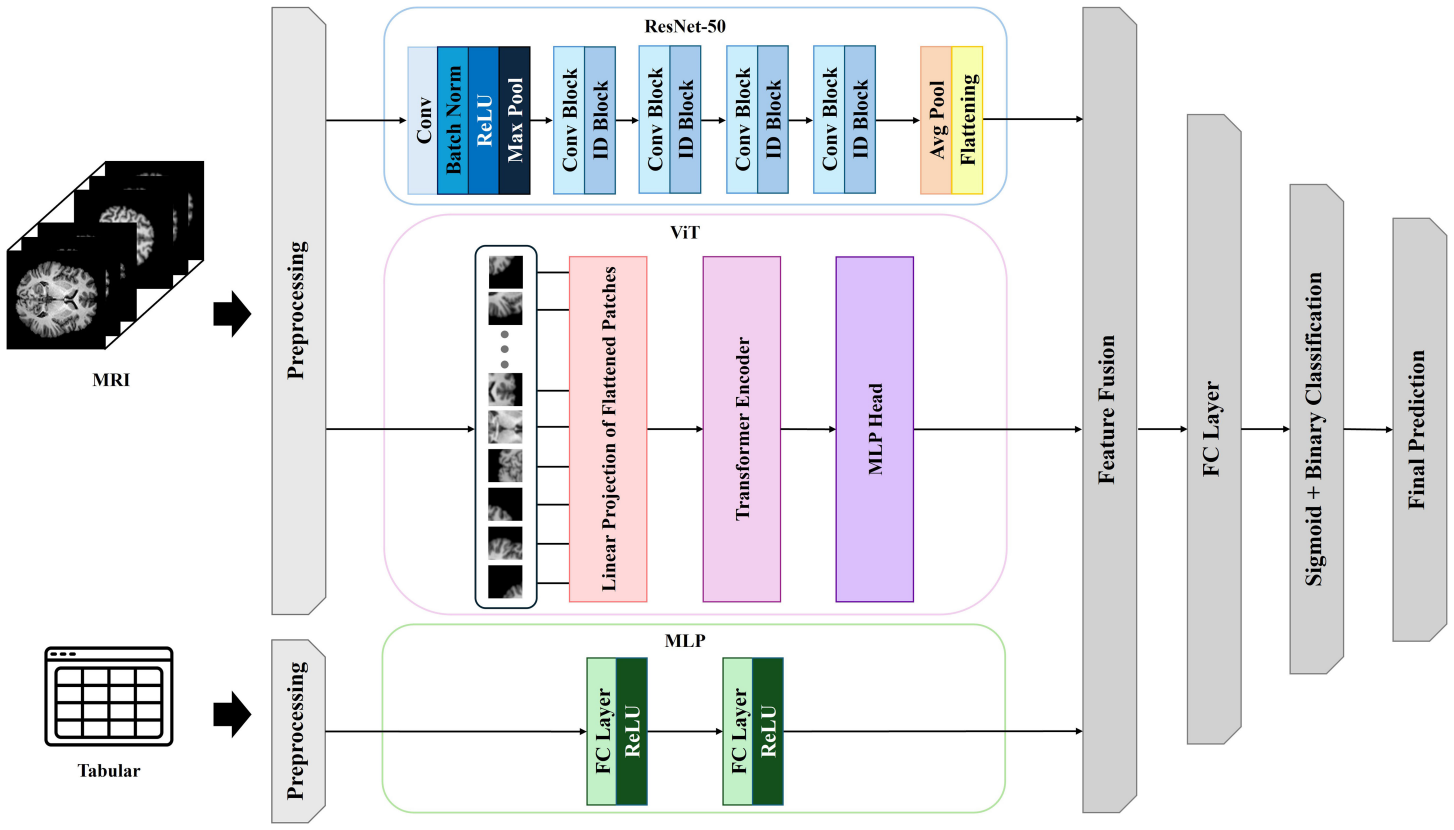
Multimodal deep learning architecture integrating neuroimaging and clinical data for alcohol use disorder risk prediction.

To predict alcohol use disorder (AUD) risk in firefighters, we developed a multimodal deep learning framework that integrates structural magnetic resonance imaging (MRI) with clinical and neuropsychological data. The framework comprised three parallel processing branches: a convolutional neural network (CNN) based on ResNet-50 for local morphological feature extraction from MRI images (30), a Vision Transformer (ViT) module for global contextual representation of neuroanatomical structures (31), and a multilayer perceptron (MLP) for incorporating clinical and neuropsychological variables (32). **Figure 2** provides a schematic overview of the deep learning architecture, illustrating the parallel processing of MRI images and clinical variables through the ResNet-50, Vision Transformer, and MLP modules, the subsequent feature concatenation, and the final classification layer.

For the MRI input stream, 80 axial two-dimensional slices per participant were fed into a pretrained ResNet-50 model to derive hierarchical local features. The resulting feature maps underwent average pooling and flattening operations to produce compact representations. In parallel, the same MRI slices were input into the ViT module via patch-based linear embedding. The ViT extracted long-range spatial dependencies and global structural context across brain regions (33). This dual-path design allowed for the concurrent extraction of both local and global representations from the neuroimaging data.

Simultaneously, clinical and neuropsychological features comprising age, sex, AUDIT score, Grooved Pegboard Test completion times (dominant and non-dominant hand), and Trail Making Test A and B durations were input into an MLP consisting of two fully connected layers with ReLU activations, yielding latent clinical representations. The outputs from the ResNet-50, ViT, and MLP branches were concatenated into a unified feature vector, which was passed through a fully connected layer with a sigmoid activation function to generate a binary prediction of AUD risk. Model training was conducted using the Adam optimizer with an initial learning rate of 0.001, a batch size of 32, and a maximum of 100 training epochs. Early stopping was applied with a patience threshold of 10 epochs based on validation loss. To mitigate overfitting and improve generalizability, dropout regularization (dropout rate = 0.5) was applied to fully connected layers, and batch normalization was incorporated after each convolutional block.

Model evaluation was performed using stratified three-fold cross-validation with participant-level data partitioning to ensure independence between training and validation sets. Performance was assessed based on accuracy, area under the receiver operating characteristic curve (AUROC), sensitivity, and specificity. Statistical differences in AUROC between model configurations were evaluated using DeLong’s test (34). All models were implemented in PyTorch (v1.10) and trained on an NVIDIA RTX A6000 GPU.

### 2.7 Model evaluation and statistical analysis

Model performance was evaluated using stratified threefold cross-validation with participant-level data partitioning to ensure independence between training and validation sets. Performance metrics included accuracy, area under the receiver operating characteristic curve (AUROC), precision, and recall. The AUROC was the primary metric due to its robustness to class imbalance. Confidence intervals (95% CI) for AUROC were estimated via bootstrapping (1,000 iterations).

Comparative analyses assessed the multimodal model against unimodal models (MRI-only, clinical-only). Between-model differences in AUROC were tested using DeLong’s method, which accounts for the correlation inherent to paired ROC curves evaluated on the same cases (34). Calibration was evaluated with reliability (calibration) curves to assess agreement between predicted probabilities and observed outcomes, and decision curve analysis was used to quantify net clinical benefit across threshold probabilities relevant to occupational screening. All preprocessing statistics, any calibration fits, and threshold selection were performed within training folds only and applied to the corresponding validation folds to avoid information leakage. Feature importance was analyzed using integrated gradients to enhance interpretability by identifying influential neuroanatomical and clinical inputs contributing to predictions (35). Statistical analyses were conducted using Python (version 3.9), Scikit-learn (version 1.0), and SciPy (version 1.7).

### 2.8 SHapley Additive exPlanations

Feature importance analysis of clinical variables was conducted using SHAP methodology with an XGBoost classifier (36) trained on clinical features comprising age, sex, AUDIT scores, Grooved Pegboard Test completion times, and Trail Making Test durations. SHAP values were computed using TreeExplainer, which leverages the tree structure for efficient Shapley value calculation (37). Global feature importance was quantified through mean absolute SHAP values across the cohort, providing interpretable measures of each variable’s contribution to risk prediction. Statistical significance of feature contributions was evaluated using permutation-based null hypothesis testing with multiple comparison correction.

### 2.9 Gradient-weighted class activation mapping

Gradient-weighted Class Activation Mapping (Grad-CAM) was employed to elucidate the spatial localization of discriminative neuroanatomical features contributing to alcohol use disorder risk classification (38). This interpretability methodology generates visual explanations by computing the gradient of the predicted class score with respect to the final convolutional layer activations, thereby identifying brain regions that maximally influence the classification decision. The analysis targeted the terminal convolutional layers of each architecture, which preserve spatial resolution while encoding high-level semantic features. The importance weights α_k_^c^ for each feature map k with respect to target class c were computed through gradient backpropagation:


$$a_{k}^{c}=\left(\frac{1}{Z}\right)\sum_{i}\sum_{j}\frac{\partial y^{c}}{\partial A_{ij}^{k}}$$

where y^c^ denotes the class score, A^k^_ij_ represents the activation at spatial location (i,j) in feature map k, and Z normalizes by spatial dimensions. The final class-discriminative localization map was generated through weighted combination of forward activation maps:


$$L_{Grad-CAM}^{c}=ReLU\left(\sum_{k}a_{k}^{c}A^{k}\right)$$

The resulting coarse-grained heatmaps underwent bilinear interpolation to match the original image resolution (224×224 pixels) and were superimposed on the corresponding MRI slices with a transparency coefficient of 0.6 to facilitate anatomical interpretation. Visualizations were generated for a randomly selected subset of 50 participants per risk category to assess spatial consistency of learned features. Dice similarity coefficients quantified the spatial overlap of activation patterns across participants, while occlusion sensitivity analysis validated the causal importance of identified regions by measuring classification confidence degradation upon masking the upper quintile of activation intensities. Given these implementation details, we briefly justify our choice of localization method. We selected Grad-CAM after considering alternative feature-localization techniques because it is class-discriminative, CNN-architecture agnostic, and computationally efficient for 2D multi-slice MRI. Unlike vanilla saliency, which is high-variance and visually noisy, Grad-CAM yields stable, coarse-to-mid-scale heatmaps aligned with the target class. Integrated Gradients requires a baseline and path integral whose choice is non-trivial for T1 intensity scales and can introduce baseline-dependent artifacts, whereas Grad-CAM avoids a baseline choice while remaining faithful to score–gradient information. Occlusion/perturbation and LIME/SHAP image explanations impose heavy sampling costs and design choices (e.g., patch size, superpixels) that scale poorly to ~80 slices per subject (37, 39). Transformer attention maps are not inherently class-specific and may not reflect decision-critical evidence, whereas Grad-CAM is explicitly class-discriminative. In medical imaging, Grad-CAM’s regional localization aligns with radiological reading practices, enabling transparent overlays on axial slices and cohort-level aggregation without re-training. To address known limitations (resolution tied to the last conv layer), we performed sanity checks (parameter randomization and slice-wise ablation) and report both representative and aggregated maps (40).

## 3 Results

**Table 2** summarizes the comparative performance of multiple predictive models for alcohol use disorder (AUD) risk classification, including clinical-only, neuroimaging-only, multi-scale image integration, and multimodal models integrating neuroimaging and clinical data. Metrics include accuracy, area under the receiver operating characteristic curve (AUROC), precision, and recall were evaluated using stratified threefold cross-validation to ensure robust estimates.

**Table 2:** Performance comparison of alcohol use disorder risk prediction models.

Model	Accuracy	AUROC	Precision	Recall
Clinical only				
Logistic Regression	0.6253	0.5773	0.6117	0.6432
MLP	0.5637	0.5436	0.5521	0.5748
Random Forest	0.5487	0.5388	0.5294	0.5562
XGBoost	0.4857	0.4795	0.4783	0.4620
Image only				
ResNet50	0.6153	0.5773	0.6089	0.6241
EfficientNet-B0	0.5967	0.5648	0.5891	0.6034
ViT	0.5457	0.5395	0.5412	0.5480
DeiT	0.5037	0.5236	0.5001	0.5076
Multi-scale image				
ResNet50 + ViT	0.6354	0.6187	0.6213	0.6495
ResNet50 + DeiT	0.5833	0.5325	0.5702	0.5894
EfficientNet-B0 + ViT	0.5902	0.5869	0.5820	0.5981
EfficientNet-B0+ DeiT	0.5627	0.5398	0.5514	0.5739
Multimodal (image + clinical)				
ResNet50 + ViT + MLP	**0.7988**	**0.7965**	**0.7836**	**0.8124**
ResNet50 + ViT + LR	0.6887	0.6726	0.6752	0.6989
ResNet50 + DeiT + MLP	0.7726	0.7563	0.7590	0.7852
EfficientNet-B0+ DeiT + LR	0.6429	0.6854	0.6381	0.6587

Performance metrics (Accuracy, AUROC, Precision, Recall) for alcohol use disorder risk prediction models across four architectural categories: clinical data only models, neuroimaging only architectures, multi scale image integration approaches, and multimodal frameworks combining neuroimaging with clinical variables. Bold values indicate the highest performance metrics across all evaluated models.

Among clinical-only models, logistic regression yielded the best performance, achieving an accuracy of 62.53%, AUROC of 57.73%, precision of 61.17%, and recall of 64.32%. Other clinical models, including multilayer perceptron (MLP), random forest, and XGBoost, demonstrated lower classification accuracy and area under the curve (AUC), with AUROCs ranging from 47.95% to 54.36%.

In the neuroimaging-only condition, the ResNet-50 model outperformed other architectures such as EfficientNet-B0, Vision Transformer (ViT), and Data-efficient Image Transformer (DeiT), achieving an AUROC of 57.73% and an accuracy of 61.53%. The ViT and DeiT models yielded AUROCs of 53.95% and 52.36%, respectively, suggesting that these transformer-based models did not surpass the convolutional baseline in unimodal imaging tasks.

Combining multiple image architectures slightly improved performance. The ResNet-50 + ViT hybrid configuration achieved the highest AUROC (61.87%) and accuracy (63.54%) within the multi-scale image category. Nonetheless, performance remained suboptimal compared to multimodal approaches.

The multimodal frameworks that integrated both neuroimaging and clinical data demonstrated significant improvements in predictive performance. The optimal configuration consisted of a fusion architecture incorporating ResNet-50, ViT, and an MLP for clinical variables, which achieved an accuracy of 79.88%, AUROC of 79.65%, precision of 78.36%, and recall of 81.24%. This performance represents a 17.35 percentage point improvement in accuracy and a 21.92 percentage gain in AUROC over the best clinical-only model (logistic regression), thereby providing compelling evidence for the additive benefit of multimodal integration. Other multimodal variants such as ResNet-50 + DeiT + MLP and ResNet-50 + ViT + logistic regression also showed superior performance relative to unimodal baselines but did not match the top-performing model.

Statistical comparison of AUROC values using DeLong’s test confirmed that the multimodal ResNet-50 + ViT + MLP model significantly outperformed both clinical-only and image-only models (p< 0.001).

**Figure 3:**
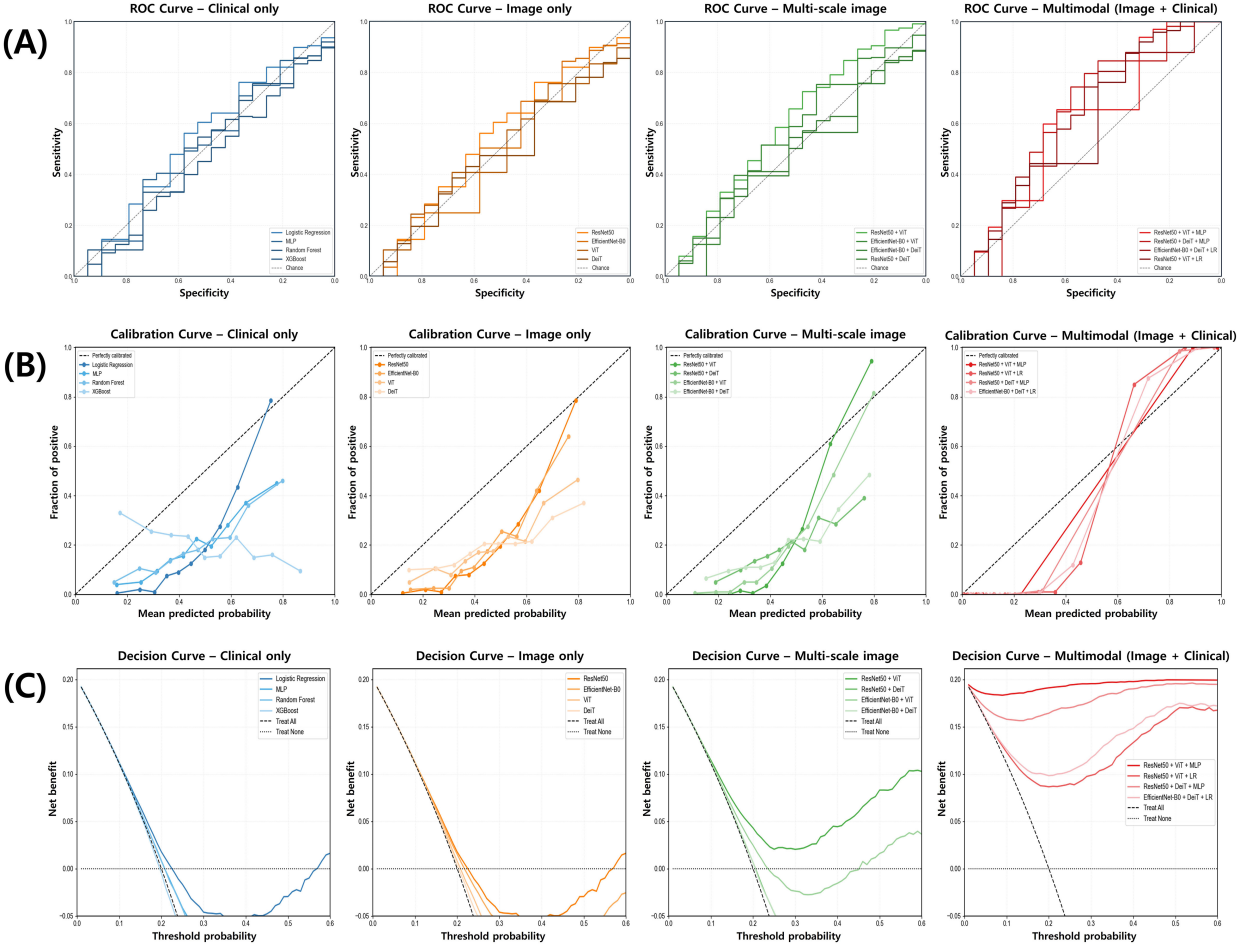
Comparison of model performance for alcohol use disorder risk prediction across data modalities. **(A)** Receiver operating characteristic (ROC) curves illustrate the discriminative performance of clinical-only, image-only, multi-scale image, and multimodal models, with the multimodal model showing the highest area under the curve (AUC). **(B)** Calibration curves compare predicted versus observed probabilities, demonstrating superior calibration in the multimodal model. **(C)** Decision curve analysis indicates that the multimodal model provides the greatest net clinical benefit across a range of threshold probabilities.

**Figure 3** provides a visual representation of model performance across three complementary dimensions. The ROC curves (**Figure 3A**) demonstrate a clear separation between the multimodal architecture and other modeling approaches, with the multimodal curve exhibiting a substantially greater area under the curve (AUC). The multi-scale image model shows intermediate discriminative capacity, positioned between the multimodal framework and the unimodal approaches, which demonstrate comparable but less robust discriminative performance.

Calibration curves (**Figure 3B**) reveal that the multimodal approach aligns more closely with the ideal calibration line compared to alternative models. Clinical-only and neuroimaging-only approaches exhibit noticeable deviations from optimal calibration, particularly in lower probability regions where systematic overestimation is visually apparent.

Decision curve analysis (**Figure 3C**) illustrates that the multimodal framework provides a consistently positive net benefit across a broader range of threshold probabilities relative to other modeling strategies. In contrast, clinical-only and neuroimaging-only approaches show diminished clinical utility at higher threshold values, whereas the multimodal approach maintains its net benefit across the full probability spectrum. These visual assessments corroborate the quantitative findings presented in **Table 2**, further supporting the enhanced predictive capability achieved through multimodal integration.

**Figure 4:**
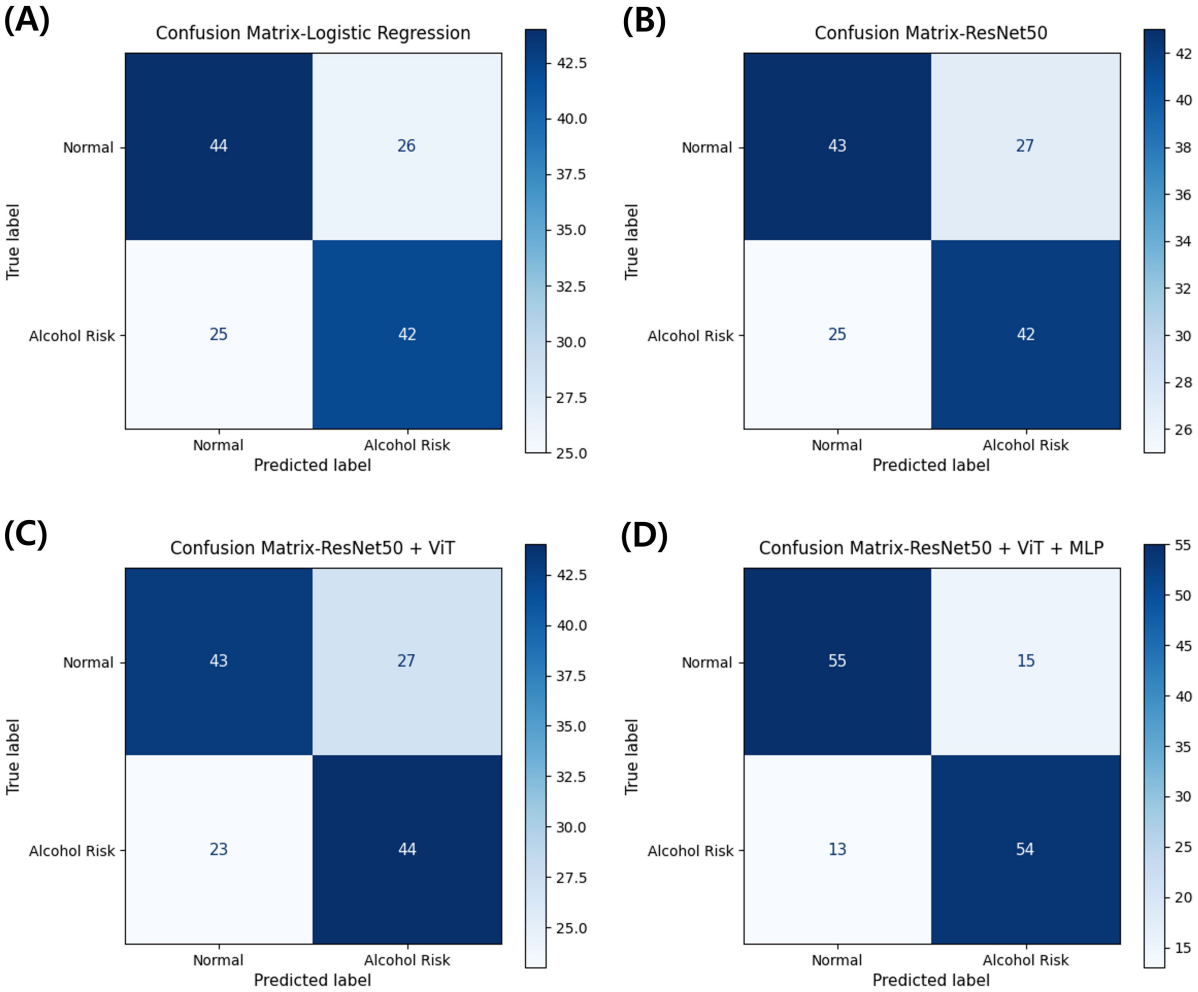
Confusion matrices comparing classification performance across model architectures for alcohol use disorder risk prediction. **(A)** Clinical-only model using logistic regression. **(B)** Image-only model using ResNet-50. **(C)** Multi-scale image model combining ResNet-50 and Vision Transformer. **(D)** Multimodal model integrating ResNet-50, Vision Transformer, and clinical variables through MLP. Values represent the number of participants classified in each category from stratified 3-fold cross-validation.

**Figure 4** shows confusion matrices for representative models (clinical-only Logistic Regression; image-only ResNet-50; multi-scale image ResNet-50 + ViT; multimodal ResNet-50 + ViT + MLP). The multimodal model yielded TN = 55, FP = 15, FN = 13, TP = 54. The clinical-only model produced TN = 44, FP = 26, FN = 25, TP = 42. The image-only model produced TN = 43, FP = 27, FN = 25, TP = 42. The multi-scale image model produced TN = 43, FP = 27, FN = 23, TP = 44. Overall, the multimodal configuration simultaneously reduced both FP and FN relative to the other approaches, indicating a more favorable error profile for occupational screening.

To examine the feature extraction patterns of neuroimaging-only models, we performed gradient-weighted class activation mapping (Grad-CAM) analysis on both ResNet-50 and EfficientNet-B0 architectures. **Supplementary Figure S1** displays representative Grad-CAM visualizations of axial brain slices from randomly selected participants processed through these convolutional neural network models. The Grad-CAM activations from both architectures exhibited substantial spatial heterogeneity across slices. Activation intensities showed irregular distributions, with discrete focal hotspots in some regions and diffuse low-intensity patterns across broader anatomical areas. Both ResNet-50 and EfficientNet-B0 revealed no systematic concentration within specific neuroanatomical structures, with high-intensity regions appearing stochastically distributed across cortical and subcortical territories. Peak activation values varied markedly across slices and architectures, ranging from isolated punctate foci to broad activation zones encompassing multiple anatomical regions.

To further justify the use of Grad-CAM over alternative feature localization methods, we additionally applied Vanilla Saliency (**Supplementary Figure S2**), Integrated Gradients (**Supplementary Figure S3**), and Occlusion Sensitivity (**Supplementary Figure S4**). Compared with Grad-CAM, Vanilla Saliency and Integrated Gradients produced noisy, low-contrast attribution maps with limited anatomical interpretability, consistent with known limitations of these gradient-based approaches when applied to structural MRI data. Occlusion Sensitivity yielded block-like activation patterns resulting from the perturbation grid but failed to delineate neuroanatomically meaningful regions in a stable manner. In contrast, Grad-CAM consistently generated smoother and more interpretable overlays, aligning with prior reports demonstrating its robustness and clinical plausibility in neuroimaging. These supplementary comparisons underscore the suitability of Grad-CAM as the primary visualization approach in this study.

These visualization outputs corroborate the quantitative performance metrics observed for the neuroimaging-only models (ResNet-50 AUROC: 57.73%, accuracy: 61.53%; EfficientNet-B0 AUROC: 56.54%, accuracy: 60.82%). The absence of consistent activation patterns across the randomly sampled cases in both architectures provides empirical evidence for the limited feature extraction capability of image-only models in this AUD risk prediction task.

Feature importance analysis of the clinical variables was conducted using SHapley Additive exPlanations (SHAP) to quantify individual feature contributions to the multimodal model predictions. **Supplementary Figure S2** presents the SHAP value distributions for six clinical features incorporated in the optimal multimodal framework.

The SHAP analysis revealed differential feature contributions across the clinical variable set. Sex demonstrated the most pronounced positive impact on model predictions, with SHAP values ranging from approximately -0.05 to +0.45, exhibiting a strong rightward skew. Non-dominant hand Grooved Pegboard completion times (GP_nondom_sec_adj) displayed bidirectional effects with SHAP values distributed between -0.30 and +0.25, indicating variable contributions to risk prediction depending on individual performance levels.

Age exhibited a balanced distribution of SHAP values spanning -0.25 to +0.20, with the majority of instances clustering near zero. Dominant hand Grooved Pegboard performance (GP_dom_sec_adj) showed similar bidirectional patterns with values ranging from -0.20 to +0.15. Trail Making Test Part A completion times (TrailA_time_adj) demonstrated moderate feature importance with SHAP values between -0.15 and +0.15. Trail Making Test Part B completion times (TrailB_time_adj) yielded the most concentrated distribution around zero, with limited outliers extending to ±0.20, suggesting minimal direct contribution to prediction outcomes in the multimodal context.

These quantitative feature attribution results complement the multimodal model performance metrics, providing mechanistic insights into the relative contributions of individual clinical variables within the integrated predictive framework.

## 4 Discussion

The multimodal deep learning framework demonstrated superior classification performance, validating the synergistic integration of structural neuroimaging with clinical assessments for AUD risk stratification in firefighters. The principal findings encompass: (1) The synergistic combination of ResNet-50 and Vision Transformer architectures facilitates complementary extraction of local morphological features and global spatial dependencies from structural MRI data, obviating computationally intensive functional connectivity analyses; (2) Integration of standardized neuropsychological assessments, specifically the Grooved Pegboard Test and Trail Making Test, provides functional neurological proxies that partially compensate for the absence of task-based or resting-state functional MRI data; (3) The multimodal framework demonstrates a 17.35 percentage point improvement in classification accuracy relative to clinical-only models, substantiating the discriminative value of structural neuroimaging when appropriately integrated with behavioral metrics; (4) Feature importance analysis identifies sex as the predominant clinical predictor, followed by motor coordination measures, elucidating potential sex-specific vulnerability patterns within this occupational cohort; (5) The model maintains robust calibration across probability thresholds, suggesting clinical applicability for risk stratification without the operational complexity inherent to functional neuroimaging protocols.

### 4.1 Comparative analysis with extant literature

**Table 3** provides a comprehensive summary of recent multimodal deep learning approaches for psychiatric disorder prediction, contextualizing our findings within the broader landscape of neuroimaging-based classification studies. Contemporary neuroimaging investigations have consistently demonstrated the superiority of multimodal approaches combining structural MRI, functional task-based MRI, and resting-state functional connectivity in psychiatric classification tasks (46). However, the multimodal framework presented herein, achieving 79.88% accuracy (AUROC: 0.795) through structural MRI and clinical assessment integration alone, demonstrates competitive performance relative to architectures incorporating functional neuroimaging. A recent triple-modality integration study (42) (sMRI + fMRI + SNP) yielded 79.01% accuracy in schizophrenia classification (n=492), with individual modalities contributing differentially (sMRI: 66.33%, fMRI: 75.29%, SNP: 57.06%). The marginal improvement from sMRI baseline to multimodal integration (13.68 percentage points) must be contextualized against substantially increased acquisition complexity and computational burden. A comprehensive investigation utilizing an extensive neuroimaging battery comprising 119 alcohol-dependent patients and 97 controls revealed that while multimodal integration yielded optimal classification performance, the investigators concluded that “in terms of direct clinical applicability, currently the most realistic neuroimaging-based classifier for AD may be unimodal based on structural MRI and grey-matter density specifically” (46), citing the temporal demands and analytical complexity of functional MRI protocols. This empirical observation corroborates our methodological decision to prioritize T1-weighted structural MRI as the primary neuroimaging modality.

**Table 3:** Comparative summary of multimodal deep learning approaches for psychiatric disorder prediction.

Study	Modalities	Target disease	Accuracy	AUROC	Sample size	Sample characteristic
Zheng et al. (41)	sMRI + fMRI	MDD	75.2%	0.808	2319	Control MDD
Kanyal et al. (42)	sMRI + fMRI + SNP	SZ	79.01%	–	492	Control SZ
Zhu et al. (43)	sMRI (3D) + fMRI	AUD	67.4% -90.5%	–	92	Control AUD
Vergara et al. (44)	fMRI	AUD	–	0.79	102	Control AUD
Kamarajan et al. (45)	fMRI	AUD	76.67%	0.93	60	Control AUD Male participants only
Guggenmos et al. (46)	sMRI + fMRI	AUD	79.3%	–	216	Control AUD
Ours	**sMRI (2D)** + **Clinical**	**AUD**	**79.88%**	**0.795**	**689**	**Occupational (Firefighters)**

Reported values are taken from the cited papers; numbers are not directly comparable across studies because of differences in datasets, label definitions (diagnosis vs. risk), cohort composition, scanners/protocols, and evaluation procedures (cross-validation vs. held-out tests). When a study reported multiple results, we list a representative value or a range; “-” indicates the metric was not reported.

Modalities: sMRI, T1-weighted structural MRI; fMRI, functional MRI (resting or task-based as reported); SNP, single-nucleotide polymorphisms.

Target disease: MDD, major depressive disorder; SZ, schizophrenia; AUD, alcohol use disorder.

Sample size is the total N analyzed in each study; Sample characteristic summarizes comparison groups (e.g., control vs. disorder, sex restrictions).

Ours denotes an occupational firefighter cohort and a multimodal model using sMRI (2D axial slices) + clinical variables without fMRI; results are averaged over stratified 3-fold, subject-wise cross-validation.

Bold values represent results from the current study.

Previous investigations employing resting-state functional connectivity have reported classification accuracies ranging from 61.53% to 76.67% for discriminating alcohol-dependent individuals from controls (43, 44). Direct comparison with AUD-focused investigations reveals our framework’s competitive performance despite methodological parsimony. A recent study (44)reported resting-state fMRI yielding AUROC 0.79 (n=102), comparable to our 0.795 despite utilizing computationally intensive connectivity analyses. This equivalence challenges assumptions regarding the superior discriminative capacity of functional imaging for AUD detection. Similarly, another investigation (46) demonstrated that dual neuroimaging modality integration (sMRI + fMRI) achieved 79.3% accuracy in AUD classification (n=216), representing merely 2.7 percentage points improvement over single modality (76.6%) which represents a limited enhancement that raises critical questions regarding the cost-effectiveness of functional imaging protocols in occupational screening contexts. Random Forest classification leveraging functional connectivity within the Default Mode Network combined with neuropsychological measures achieved 76.67% accuracy (45), necessitating extensive preprocessing pipelines and network-level analytical frameworks. Recent work (45) reported fMRI-based classification achieving 76.67% accuracy (AUROC: 0.93) in a male-exclusive cohort (n=60). Our superior accuracy (79.88%) in a substantially larger sample (n=689) with mixed-gender composition suggests that structural alterations combined with behavioral assessments may provide greater discriminative capacity than functional connectivity alone in occupational populations. Resting-state connectivity features have demonstrated capacity to explain 33% of variance in Alcohol Use Disorders Identification Test (AUDIT) scores (47), though such models required acquisition of multiple functional MRI sequences including monetary incentive delay and face-matching paradigms alongside resting-state protocols.

Multimodal data integration approaches in psychiatric research demonstrate methodological advantages comparable to our neuroimaging-clinical framework. A recent VR-based study (48) developed machine learning models utilizing acoustic and physiological features VR exposure sessions for social anxiety disorder, achieving an AUROC of 0.852 with CatBoost for Social Phobia Scale prediction using multimodal features (n=132 samples from 25 participants). Notably, their analysis revealed that acoustic features (AUROC: 0.788) substantially outperformed physiological features alone (AUROC: 0.626) for anxiety symptom prediction, with multimodal integration yielding superior classification performance across multiple anxiety domains. While their VR-based approach differs methodologically from our structural neuroimaging framework, the 7.26 percentage point improvement from physiological to multimodal features (compared to our 17.35 percentage point improvement from clinical to multimodal) highlights the consistent benefit of cross-modal integration in psychiatric risk stratification. Their findings that acoustic biomarkers captured more discriminative information than physiological responses during anxiety-inducing scenarios parallels our observation that targeted neuropsychological assessments provide critical functional anchoring for structural alterations.

The classification performance of our T1-weighted structural MRI multimodal approach (79.88% accuracy) demonstrates favorable comparison with functional connectivity-based methodologies while offering considerable practical advantages regarding acquisition efficiency and computational parsimony. Our framework’s 17.35 percentage point improvement from clinical-only baseline (62.53%) substantially exceeds the incremental gains observed when adding neuroimaging to clinical data reported previously (46), suggesting that targeted neuropsychological assessments may capture variance typically attributed to functional connectivity measures. A systematic review examining machine learning applications in AUD reported neuroimaging-based algorithms achieving sensitivity ranging from 90-99.99% and specificity from 82-99.97% (14); however, these exceptional performance metrics were predominantly observed in investigations combining multiple imaging modalities. One study (43)reported 3D sMRI + fMRI combination achieving accuracy ranging from 67.4% to 90.5% (n=92). The substantial variability suggests potential overfitting in small samples, emphasizing the importance of our larger cohort (n=689) for robust generalization estimates. Beyond structural neuroimaging approaches, recent advances in machine learning applications for mental health monitoring in first responders provide complementary perspectives on psychological distress prediction. A proof-of-concept investigation (49) developed predictive models for posttraumatic stress injuries (PTSI) utilizing intensive longitudinal data from 274 Montreal firefighters monitored biweekly across 12 weeks. The study implemented four distinct machine learning algorithms (logistic regression, support vector classifier, extreme gradient boosting) trained on temporal sequences of standardized psychological assessments (PHQ-9, GAD-7, PCL-5) and psychosocial variables (occupational stress, social support, coping strategies). The optimal model configuration, employing extreme gradient boosting with three lagged measurement timepoints and comprehensive feature sets, achieved 94% classification accuracy (AUC = 0.93, sensitivity = 0.61, specificity = 0.97). Several methodological contrasts with the present investigation merit consideration. The documented PTSI prevalence, fluctuating between 6.9% and 10.6% across assessment intervals with cumulative incidence of 19.7%, represents substantially lower psychopathology rates than our observed AUD risk prevalence of 56.9%, potentially attributable to differential diagnostic thresholds between acute stress-related symptomatology and chronic alcohol use vulnerability. Feature importance analyses identified lagged PHQ-9 scores collected 2 and 6 weeks prior to target assessment as dominant predictors (19% and 10% relative importance respectively), with GAD-7 and PCL-5 scores contributing secondarily, while demographic variables (age >46 years, work experience >21 years) demonstrated minimal predictive value. This hierarchical pattern corresponds with our SHAP-derived feature attributions wherein neuropsychological performance metrics superseded demographic characteristics. The temporal dependency of predictive accuracy, wherein model performance systematically improved from single-timepoint (accuracy range: 0.81-0.91) to three-timepoint configurations (accuracy range: 0.82-0.94), underscores the critical importance of longitudinal symptom trajectories in psychiatric risk modeling. These convergent findings across distinct methodological paradigms substantiate the superiority of multimodal, temporally-informed approaches over cross-sectional univariate assessments. Integration of periodic structural neuroimaging for baseline vulnerability characterization with continuous smartphone-based symptom monitoring could potentially optimize early intervention strategies through synthesis of stable neurobiological markers and dynamic clinical trajectories. Our approach achieves minimal sacrifice in predictive accuracy while greatly reducing acquisition time, computational burden, and technical expertise requirements (49).

Previous investigations utilizing isolated structural MRI modalities have provided valuable performance benchmarks, with grey matter density analysis achieving 65% classification accuracy in comprehensive multimodal comparisons (46). The present study builds upon these findings by demonstrating that augmenting structural neuroimaging with targeted neuropsychological assessments yields enhanced discriminative capacity (79.88% accuracy), consistent with theoretical frameworks positing synergistic information capture across neurobiological and behavioral domains. The 17.35 percentage point improvement from clinical baseline reflects fundamental complementarity rather than simple feature concatenation: structural neuroimaging captures cumulative morphological alterations reflecting chronic alcohol exposure, providing stable biomarkers of neurotoxic burden, while neuropsychological performance offers dynamic functional readouts of neural system integrity sensitive to subclinical impairments. This performance differential underscores the critical importance of incorporating standardized neuropsychological assessments to compensate for the absence of functional connectivity information. Recent investigations have emphasized that machine learning algorithms provide valuable tools for quantifying large-scale network differences in AUD (44); however, our results suggest that morphological features combined with targeted clinical assessments achieve comparable discriminative capacity.

The firefighter population presents unique challenges for AUD prediction modeling. While general population studies have examined heterogeneous samples characterized by varied substance use histories and psychiatric comorbidities (43), our cohort’s occupational homogeneity and elevated baseline risk necessitated tailored analytical approaches. With n=689, our investigation represents the second-largest cohort among reviewed studies [following a recent MDD study (41): n=2319], providing robust statistical power while maintaining occupational homogeneity. While T1-weighted structural sequences and fMRI share similar acquisition times (5–10 minutes each), the critical distinction lies in post-processing complexity. Functional MRI necessitates sophisticated preprocessing pipelines encompassing motion correction, temporal filtering, spatial smoothing, and connectivity analysis, extending analysis time from hours to days. Additionally, fMRI’s heightened motion sensitivity increases data attrition rates, compromising practicality for large-scale screening initiatives. Previous occupational cohort investigations remain limited, constraining direct performance comparisons. Nevertheless, the effective classification achieved without functional MRI suggests that structural alterations and behavioral manifestations may exhibit enhanced discriminability in high-risk occupational groups, potentially attributable to chronic stress exposure and cultural factors influencing alcohol consumption patterns. The elimination of fMRI-specific infrastructure requirements (stimulus presentation systems, synchronization hardware, specialized preprocessing software) substantially reduces implementation barriers in clinical settings, supporting the translational feasibility of our approach for occupational health surveillance.

### 4.2 Mechanistic considerations

The efficacy of our multimodal approach necessitates examination through complementary interpretability methodologies to elucidate differential contributions of neuroimaging and clinical features. Recent advances in multimodal explainable AI have demonstrated the critical importance of understanding feature interactions across modalities. A recent study achieved 94.81% accuracy using an Ensemble Optimization-enabled Explainable CNN (EO-ECNN) with multimodal data integration, highlighting the significance of interpretability in clinical applications (50). Recent comparative studies have systematically evaluated various explainability approaches for multimodal medical imaging. A large-scale experiment across four medical imaging datasets found that while attention maps from Vision Transformers generally surpass Grad-CAM in explainability, transformer-specific interpretability methods demonstrate superior performance (51). This finding underscores the importance of selecting architecture-appropriate interpretability techniques rather than applying traditional CNN-based methods to transformer architecture. Gradient-weighted Class Activation Mapping (Grad-CAM) analysis applied to unimodal neuroimaging models revealed critical insights regarding the limitations of structural MRI-only approaches. As illustrated in **Supplementary Figure S1**, Grad-CAM visualizations from both ResNet-50 (Panel A) and EfficientNet-B0 (Panel B) architectures demonstrated stochastic activation patterns across randomly selected axial brain slices. The activation maps exhibited no systematic concentration within anatomically relevant regions associated with alcohol-related neurodegeneration, instead displaying diffuse, heterogeneous patterns with focal hotspots appearing randomly across cortical and subcortical territories. The stochastic activation patterns observed through Grad-CAM analysis provide empirical evidence for the fundamental limitations of structural MRI-only approaches in detecting subtle, distributed alterations associated with AUD risk. This finding aligns with previous neuroimaging studies showing that morphological changes in early-stage AUD are often diffuse and heterogeneous, requiring behavioral anchoring for meaningful interpretation (46, 52).

This absence of neuroanatomically coherent feature extraction in image-only models provides mechanistic validation for observed performance limitations (ResNet-50: 57.73% AUROC; EfficientNet-B0: 56.54% AUROC). A comprehensive survey of explainable multimodal learning methods confirmed that such random activation patterns indicate insufficient discriminative capacity when structural alterations are subtle and distributed (53). Recent advances in transformer architecture have introduced attention visualization as a complementary interpretability approach. Studies on multimodal foundation models for anomaly detection have demonstrated that combining SHAP, Grad-CAM, and attention visualization provides more comprehensive insights than any single approach, particularly when dealing with heterogeneous medical data sources (54). These findings suggest that different XAI techniques capture complementary aspects of model behavior: spatial localization through Grad-CAM, feature importance through SHAP, and hierarchical relationships through attention mechanisms. Grad-CAM heatmaps revealed that convolutional neural networks, when constrained to structural MRI data alone, failed to converge on consistent morphological markers despite well-established volumetric alterations in alcohol-dependent populations. Peak activation intensities varied markedly between slices without correspondence to regions of established vulnerability including prefrontal cortex, hippocampus, or cerebellar structures. This stochastic behavior suggests that structural alterations alone, while present, may be insufficiently discriminative for effective classification without complementary functional or behavioral indicators.

Conversely, SHapley Additive exPlanations (SHAP) analysis of clinical variables within the optimal multimodal framework revealed hierarchical feature importance with clear mechanistic interpretability (**Supplementary Figure S2**). Sex emerged as the predominant contributor with SHAP values ranging from -0.05 to +0.45, exhibiting pronounced rightward skew indicative of male sex as a risk amplifier. This finding aligns with established sex differences in alcohol metabolism, neurotoxic vulnerability, and addiction trajectories. The prominence of biological sex as a predictor (SHAP values: -0.05 to +0.45) aligns with established epidemiological evidence showing higher AUD prevalence in male firefighters and known sex differences in alcohol metabolism and neurotoxic vulnerability (55). Non-dominant hand Grooved Pegboard performance demonstrated bidirectional effects (SHAP values: -0.30 to +0.25), suggesting that motor coordination impairments serve as sensitive indicators of subclinical neurological compromise.

The substantial performance differential between the multimodal framework (79.88% accuracy) and both clinical-only (62.53%) and imaging-only (61.53%) approaches cannot be attributed to simple additive effects of feature concatenation. Rather, this performance enhancement reflects fundamental principles of multimodal machine learning wherein complementary information sources capture distinct aspects of underlying pathophysiology (56, 57). Recent theoretical frameworks in multimodal neuroimaging emphasize that different data modalities provide non-redundant views of complex biological phenomena, with optimal integration strategies exploiting this complementarity to achieve superior discriminative power (58, 59).

The integration of neuroimaging and clinical features within our multimodal architecture leverages what has been termed “cooperative fusion” in the multimodal learning literature, wherein modalities interact synergistically to reveal patterns invisible to either modality in isolation (60). This approach aligns with recent comprehensive reviews demonstrating that transformer and CNN architectures require tailored interpretability methods to effectively capture their distinct feature extraction patterns (61, 62). Structural MRI captures static morphological alterations reflecting cumulative neurotoxic effects, while neuropsychological assessments provide dynamic functional readouts of neural system integrity. The Vision Transformer component, designed to capture global spatial dependencies, may identify distributed patterns of subtle atrophy that achieve diagnostic relevance only when contextualized by concurrent functional deficits captured through clinical assessments. This synergistic relationship aligns with recent findings demonstrating that multimodal approaches consistently outperform unimodal methods in neuropsychiatric classification tasks by capitalizing on the complementary nature of structural and functional information (63, 64).

Critically, observed performance gains cannot be explained by overfitting to clinical features or trivial demographic correlations. SHAP analysis reveals that while sex contributes significantly, motor coordination measures and other neuropsychological indicators provide substantial independent predictive value. Moreover, the failure of clinical-only models to exceed 62.53% accuracy demonstrates that behavioral assessments alone lack sufficient discriminative capacity. Similarly, poor performance of imaging-only models indicates that structural alterations, though present, require behavioral anchoring for meaningful interpretation in this at-risk but pre-clinical population.

Mechanistic insights derived from interpretability analyses have profound implications for understanding AUD vulnerability in occupational cohorts. The failure of image-only models to identify consistent neuroanatomical markers suggests that structural alterations in early-stage or at-risk individuals may be subtle, distributed, and heterogeneous, requiring behavioral anchors for meaningful interpretation. The prominence of sex and motor coordination measures in feature importance rankings indicates that integrative models capturing both biological predisposition and functional manifestation provide superior discriminative capacity. These findings support a multifactorial conceptualization of AUD risk wherein neurobiological alterations interact with demographic vulnerabilities and manifest through measurable performance deficits before clinical thresholds are reached.

### 4.3 Methodological limitations

This investigation presents several methodological constraints warranting comprehensive examination. First, the cross-sectional design fundamentally precludes causal inference regarding temporal evolution of structural brain alterations and their relationship to AUD risk. Longitudinal investigations tracking firefighters from recruitment through career progression would be essential to establish whether observed neuroanatomical variations represent predisposing vulnerabilities, consequences of occupational stress exposure, early markers of problematic alcohol use, or complex interactions among these factors. The absence of temporal data particularly limits our ability to determine whether structural alterations precede behavioral manifestations or emerge concurrently with escalating alcohol consumption.

Additionally, exclusive reliance on T1-weighted structural MRI, while strategically chosen for clinical feasibility, imposes inherent constraints on the comprehensiveness of neurobiological characterization. Resting-state functional MRI investigations have identified specific functional connectivity alterations in reward, salience, and executive control networks that differentiate individuals with AUD from controls (43, 44, 47). Our structural-only approach cannot capture these dynamic network-level disruptions, potentially missing critical neurophysiological markers of addiction vulnerability. Future iterations incorporating abbreviated resting-state protocols or task-based functional MRI targeting reward processing could enhance predictive accuracy while maintaining reasonable clinical practicality. Additionally, advanced structural imaging techniques such as diffusion tensor imaging could provide microstructural integrity measures complementing volumetric assessments.

Furthermore, the computational decision to segment three-dimensional brain volumes into two-dimensional axial slices, while reducing computational complexity and memory requirements, sacrifices spatial continuity information. Three-dimensional convolutional architectures or graph neural networks operating on whole-brain volumes could potentially capture long-range anatomical relationships and improve feature extraction, though at substantially increased computational cost. The trade-off between model sophistication and practical deployability remains a critical consideration for clinical translation.

Moreover, generalizability of our findings faces multiple constraints. The sample comprised exclusively Korean firefighters, introducing both cultural and occupational specificity that may limit applicability to other populations. Cultural variations in alcohol consumption patterns, stigma associated with help-seeking, and occupational stress exposure could influence both structural brain alterations and model performance. The marked sex imbalance (93% male) reflects firefighting workforce demographics but severely limits conclusions about female firefighters, particularly given sex differences in alcohol metabolism, vulnerability to neurotoxic effects, and addiction trajectories. Validation in diverse cultural contexts, occupational groups, and sex-balanced samples remains essential before broader implementation.

Concomitantly, several technical limitations merit consideration. The AUDIT threshold of 8 for defining at-risk status, while internationally validated, may not optimally discriminate problematic drinking patterns in high-functioning occupational cohorts where normative drinking levels differ from general populations. The relatively modest sample size (n=689), while substantial for neuroimaging studies, may limit detection of subtle subgroup differences or complex interaction effects. The absence of genetic data precludes investigation of gene-environment interactions known to influence AUD vulnerability. Missing longitudinal follow-up data prevents validation of the model’s actual predictive utility for incident AUD diagnosis or occupational impairment.

Beyond these methodological considerations, clinical implementation faces practical challenges beyond model performance. The requirement for high-resolution structural MRI limits deployment to settings with advanced imaging facilities, potentially excluding rural or resource-limited fire departments. The need for standardized neuropsychological testing by trained personnel adds operational complexity. Privacy concerns regarding neuroimaging-based occupational screening require careful ethical consideration and policy development. The potential for algorithmic bias, particularly given the homogeneous training sample, necessitates ongoing monitoring and recalibration in diverse deployment contexts.

Finally, performance ceiling effects warrant critical consideration. While the achieved 79.88% accuracy represents competitive performance relative to existing multimodal approaches, it nonetheless implies a 20.12% misclassification rate with asymmetric consequences: false positives potentially triggering unwarranted career interventions versus false negatives resulting in missed opportunities for early therapeutic engagement. Furthermore, the absence of longitudinal follow-up data fundamentally constrains clinical interpretation; the model’s capacity to predict incident AUD diagnoses, trajectory of symptom progression, or subsequent occupational impairment remains empirically undefined. This temporal limitation restricts current applicability to cross-sectional risk stratification rather than prospective prediction, highlighting the imperative for longitudinal validation studies to establish true predictive validity and optimal screening intervals.

### 4.4 Implications for occupational health screening

The demonstrated feasibility of achieving robust AUD risk classification using structural MRI represents a significant advancement for occupational health surveillance in high-risk professions. Traditional screening paradigms relying on self-report instruments face systematic limitations in emergency response populations where cultural valorization of stoicism, occupational stigma, and career preservation concerns suppress accurate disclosure of alcohol-related problems (2, 12). The integration of objective neurobiological markers derived from structural neuroimaging circumvents these reporting biases while maintaining classification performance comparable to more complex multimodal approaches.

Implementation within existing occupational health frameworks requires consideration of both technical infrastructure and organizational factors. Fire departments conducting periodic comprehensive medical evaluations could incorporate T1-weighted structural MRI protocols, though the total examination time of 30–60 minutes represents a substantial logistical consideration. While the T1-weighted sequence itself requires only 5–10 minutes of actual acquisition time, the complete imaging protocol including patient preparation, positioning, and safety procedures necessitates dedicated scheduling within occupational health assessments. The demonstrated predictive value of combining neuroimaging with standardized neuropsychological assessments suggests that brief cognitive testing batteries could enhance screening accuracy without requiring specialized neuropsychological expertise. Automated analysis pipelines utilizing the validated deep learning architecture could provide rapid risk stratification post-acquisition, enabling occupational health physicians to prioritize intervention resources toward highest-risk individuals.

To address feasibility concerns, a tiered implementation strategy could optimize resource utilization while maintaining screening effectiveness. Initial deployment could target high-risk subpopulations identified through traditional screening tools (AUDIT scores ≥ 15) or those with documented occupational incidents, thereby concentrating MRI resources on individuals with greatest clinical need. As infrastructure develops and costs decrease, screening criteria could progressively expand to encompass broader firefighter populations. This phased approach aligns with successful precedents in occupational health screening, where targeted protocols for high-risk workers preceded universal implementation (65).

Critical ethical considerations must guide translation from research findings to occupational screening practices. Clear delineation between probabilistic risk assessment and clinical diagnosis remains essential to prevent discriminatory practices while maximizing preventive potential. Screening protocols should emphasize early intervention and support rather than punitive measures, with explicit protections ensuring that neuroimaging findings cannot adversely impact employment status without corroborating clinical evidence. Longitudinal monitoring frameworks tracking predictive validity of initial risk assessments against subsequent clinical outcomes would enable continuous algorithm refinement while building evidence for screening effectiveness.

Economic implications of neuroimaging-based screening warrant careful analysis within resource allocation frameworks. Recent economic modeling of MRI-based screening programs provides relevant benchmarks. The UK Biobank’s population neuroimaging initiative achieved per-scan costs of £264 ($330 USD) through high-volume standardization (66). Considering firefighters’ elevated AUD risk (56.9% in our cohort vs. 6.2% general population) (67) and associated costs of untreated AUD (estimated $249 billion annually in the US) (68), targeted neuroimaging screening may prove cost-effective despite initial infrastructure investments. A threshold analysis suggests that preventing one severe occupational incident per 150 screenings would offset implementation costs. Furthermore, downstream savings from prevented occupational injuries, reduced absenteeism, decreased liability exposure, and maintained operational readiness strengthen the economic justification. Insurance frameworks may require modification to recognize preventive neuroimaging in high-risk occupational cohorts as medically necessary, particularly given these demonstrated cost-benefit ratios. Future development priorities should address current limitations while enhancing clinical utility. Multicenter validation studies incorporating diverse geographical regions, departmental cultures, and demographic compositions would establish generalizability boundaries and identify population-specific calibration requirements. Integration with emerging digital biomarkers from wearable devices, sleep monitoring, and stress physiology could create comprehensive risk profiles extending beyond cross-sectional neuroimaging snapshots. Development of abbreviated screening protocols optimized for rapid deployment during routine medical evaluations could enhance feasibility while maintaining predictive accuracy.

The broader implications extend beyond firefighting to encompass other high-stress occupations with elevated substance use risk, including law enforcement, emergency medical services, and military personnel. Establishing standardized neuroimaging protocols and classification algorithms across these populations would enable comparative effectiveness research while building robust normative databases. International collaboration through occupational health networks could accelerate validation efforts while ensuring equitable access to advanced screening technologies across resource-varied settings.

## Data Availability

The dataset for this article are not publicly available due to due to ethical and privacy concerns. Requests to access the datasets should be directed to the corresponding author/s.

## References

[1] Cogan N Craig A Milligan L Mccluskey R Burns T Ptak W. ‘I’ve got no PPE to protect my mind’: understanding the needs and experiences of first responders exposed to trauma in the workplace. Eur J Psychotraumatol. (2024) 15:2395113. doi: 10.1080/20008066.2024.2395113, PMID: 39238472 PMC11382715

[2] Haddock CK Jitnarin N Caetano R Jahnke SA Hollerbach BS Kaipust CM. Norms about alcohol use among US firefighters. Saf Health At Work. (2022) 13:387–93. doi: 10.1016/j.shaw.2022.08.008, PMID: 36579011 PMC9772477

[3] Yoo JY Sarkar A Song H-S Bang S Shim G Springer C. Gut microbiome alterations, mental health, and alcohol consumption: investigating the gut–brain axis in firefighters. Microorganisms. (2025) 13:680. doi: 10.3390/microorganisms13030680, PMID: 40142574 PMC11945892

[4] Rotunda RJ Herzog J Dillard DR King E O’dare K. Alcohol misuse and correlates with mental health indicators among firefighters. Subst Use Misuse. (2025) 60:236–43. doi: 10.1080/10826084.2024.2422975, PMID: 39511710

[5] Carleton RN Afifi TO Turner S Taillieu T Duranceau S Lebouthillier DM. Mental disorder symptoms among public safety personnel in Canada. Can J Psychiatry. (2018) 63:54–64. doi: 10.1177/0706743717723825, PMID: 28845686 PMC5788123

[6] Birrell J Meares K Wilkinson A Freeston M. Toward a definition of intolerance of uncertainty: A review of factor analytical studies of the Intolerance of Uncertainty Scale. Clin Psychol Rev. (2011) 31:1198–208. doi: 10.1016/j.cpr.2011.07.009, PMID: 21871853

[7] Pretorius TB Padmanabhanunni A. The relationship between intolerance of uncertainty and alcohol use in first responders: A cross-sectional study of the direct, mediating and moderating role of generalized resistance resources. Int J Environ Res Public Health. (2025) 22:383. doi: 10.3390/ijerph22030383, PMID: 40238397 PMC11942478

[8] Hallihan H Bing-Canar H Paltell K Berenz EC. Negative urgency, PTSD symptoms, and alcohol risk in college students. Addictive Behav Rep. (2023) 17:100480. doi: 10.1016/j.abrep.2023.100480, PMID: 36698484 PMC9868323

[9] Müller CP Schumann G Rehm J Kornhuber J Lenz B. Self-management with alcohol over lifespan: psychological mechanisms, neurobiological underpinnings, and risk assessment. Mol Psychiatry. (2023) 28:2683–96. doi: 10.1038/s41380-023-02074-3, PMID: 37117460 PMC10615763

[10] Ekhtiari H Sangchooli A Carmichael O Moeller FG O’donnell P Oquendo MA. Neuroimaging biomarkers of addiction. Nat Ment Health. (2024) 2:1498–517. doi: 10.1038/s44220-024-00334-x

[11] Smith LJ Zegel M Bartlett BA Lebeaut A Vujanovic AA. Posttraumatic stress and alcohol use among first responders. In: Mental health intervention and treatment of first responders and emergency workers. Hershey, PA, USA (IGI Global): IGI Global Scientific Publishing (2020).

[12] Strudwick J Gayed A Deady M Haffar S Mobbs S Malik A. Workplace mental health screening: a systematic review and meta-analysis. Occup Environ Med. (2023) 80:469–84. doi: 10.1136/oemed-2022-108608, PMID: 37321849 PMC10423530

[13] Gonzalez DE Lanham SN Martin SE Cleveland RE Wilson TE Langford EL. Firefighter health: A narrative review of occupational threats and countermeasures. Healthcare. (2024) 12:440. MDPI. doi: 10.3390/healthcare12040440, PMID: 38391814 PMC10888326

[14] Hurtado M Siefkas A Attwood MM Iqbal Z Hoffman J. Machine learning applications and advancements in alcohol use disorder: A systematic review. medRxiv. (2022), 22276057. doi: 10.1101/2022.06.06.22276057

[15] Kinreich S Meyers JL Maron-Katz A Kamarajan C Pandey AK Chorlian DB. Predicting risk for Alcohol Use Disorder using longitudinal data with multimodal biomarkers and family history: a machine learning study. Mol Psychiatry. (2021) 26:1133–41. doi: 10.1038/s41380-019-0534-x, PMID: 31595034 PMC7138692

[16] Smucny J Shi G Davidson I. Deep learning in neuroimaging: overcoming challenges with emerging approaches. Front Psychiatry. (2022) 13:912600. doi: 10.3389/fpsyt.2022.912600, PMID: 35722548 PMC9200984

[17] Caplan B Mendoza JE. Edinburgh handedness inventory. In: Encyclopedia of clinical neuropsychology. New York, NY, USA: Springer (2011).

[18] Buard I Yang X Kaizer A Lattanzio L Kluger B Enoka RM. Finger dexterity measured by the Grooved Pegboard test indexes Parkinson’s motor severity in a tremor-independent manner. J Electromyography Kinesiol. (2022) 66:102695. doi: 10.1016/j.jelekin.2022.102695, PMID: 36030732 PMC9836835

[19] Reitan RM. Validity of the Trail Making Test as an indicator of organic brain damage. Perceptual Motor Skills. (1958) 8:271–6. doi: 10.2466/pms.1958.8.3.271

[20] Varjacic A Mantini D Demeyere N Gillebert CR. Neural signatures of Trail Making Test performance: Evidence from lesion-mapping and neuroimaging studies. Neuropsychologia. (2018) 115:78–87. doi: 10.1016/j.neuropsychologia.2018.03.031, PMID: 29596856 PMC6018614

[21] Babor TF Higgins-Biddle JC Saunders JB Monteiro MG. The alcohol use disorders identification test. World Health Organization, Geneva, Switzerland (2001).

[22] Lundin A Hallgren M Balliu N Forsell Y. The use of alcohol use disorders identification test (AUDIT) in detecting alcohol use disorder and risk drinking in the general population: validation of AUDIT using schedules for clinical assessment in neuropsychiatry. Alcohol: Clin Exp Res. (2015) 39:158–65. doi: 10.1111/acer.12593, PMID: 25623414

[23] Jenkinson M Beckmann CF Behrens TE Woolrich MW Smith SM. Fsl. Neuroimage. (2012) 62:782–90. doi: 10.1016/j.neuroimage.2011.09.015, PMID: 21979382

[24] Isensee F Schell M Pflueger I Brugnara G Bonekamp D Neuberger U. Automated brain extraction of multisequence MRI using artificial neural networks. Hum Brain Mapp. (2019) 40:4952–64. doi: 10.1002/hbm.24750, PMID: 31403237 PMC6865732

[25] Rosenbloom MJ Pfefferbaum A. Magnetic resonance imaging of the living brain: evidence for brain degeneration among alcoholics and recovery with abstinence. Alcohol Res Health. (2008) 31:362., PMID: 23584010 PMC3860463

[26] Park JS. Cross-sectional Atlas of Rhesus Monkey Head: with 0.024-mm pixel size color images. Cham, Switzerland: Springer Nature (2022).

[27] Hao R Namdar K Liu L Haider MA Khalvati F. A comprehensive study of data augmentation strategies for prostate cancer detection in diffusion-weighted MRI using convolutional neural networks. J Digital Imaging. (2021) 34:862–76. doi: 10.1007/s10278-021-00478-7, PMID: 34254200 PMC8455796

[28] Cossio M. Augmenting medical imaging: a comprehensive catalogue of 65 techniques for enhanced data analysis. (2023). arXiv preprint arXiv:2303.01178.

[29] Abdollahi B Tomita N Hassanpour S. Data augmentation in training deep learning models for medical image analysis. In: Deep learners and deep learner descriptors for medical applications. Cham, Switzerland: Springer (2020).

[30] Koonce B. ResNet 50. In: Convolutional neural networks with swift for tensorflow: image recognition and dataset categorization. Cham, Switzerland: Springer (2021).

[31] Dosovitskiy A Beyer L Kolesnikov A Weissenborn D Zhai X Unterthiner T. An image is worth 16x16 words: Transformers for image recognition at scale. (2020). arXiv preprint arXiv:2010.11929.

[32] Rumelhart DE Hinton GE Williams RJ. Learning representations by back-propagating errors. nature. (1986) 323:533–6. doi: 10.1038/323533a0

[33] Arango-Argoty G Kipkogei E Stewart R Sun GJ Patra A Kagiampakis I. Pretrained transformers applied to clinical studies improve predictions of treatment efficacy and associated biomarkers. Nat Commun. (2025) 16:2101. doi: 10.1038/s41467-025-57181-2, PMID: 40025003 PMC11873189

[34] DeLong ER Delong DM Clarke-Pearson DL. Comparing the areas under two or more correlated receiver operating characteristic curves: a nonparametric approach. Biometrics. (1988) 44:837–45. doi: 10.2307/2531595 3203132

[35] Sundararajan M Taly A Yan Q. (2017). Axiomatic attribution for deep networks, in: Proceedings of the 34th International Conference on Machine Learning (ICML 2017), Proceedings of Machine Learning Research, PMLR (online open-access publisher), 70:3319–28. PMLR. Available online at: http://proceedings.mlr.press/v70/sundararajan17a.html

[36] Chen T Guestrin C. (2016). Xgboost: A scalable tree boosting system, in: Proceedings of the 22nd ACM SIGKDD International Conference on Knowledge Discovery and Data Mining (KDD '16), New York, NY, USA: Association for Computing Machinery (ACM). pp. 785–94.

[37] Lundberg SM Lee S-I. A unified approach to interpreting model predictions. Adv Neural Inf Process Syst. (2017) 30:4765–74.

[38] Selvaraju RR Cogswell M Das A Vedantam R Parikh D Batra D. (2017). Grad-cam: Visual explanations from deep networks via gradient-based localization, in: Proceedings of the IEEE International Conference on Computer Vision (ICCV 2017), Los Alamitos, CA, USA: IEEE Computer Society. pp. 618–26.

[39] Ribeiro MT Singh S Guestrin C. (2016). Why should i trust you?” Explaining the predictions of any classifier, in: Proceedings of the 22nd ACM SIGKDD International Conference on Knowledge Discovery and Data Mining (KDD '16), New York, NY, USA: Association for Computing Machinery (ACM). pp. 1135–44.

[40] Adebayo J Gilmer J Muelly M Goodfellow I Hardt M Kim B. Sanity checks for saliency maps. Adv Neural Inf Process Syst. (2018) 31:9505–15.

[41] Zheng G Zheng W Zhang Y Wang J Chen M Wang Y. An attention-based multi-modal MRI fusion model for major depressive disorder diagnosis. J Neural Eng. (2023) 20:056037. doi: 10.1088/1741-2552/ad038c, PMID: 37844568

[42] Kanyal A Mazumder B Calhoun VD Preda A Turner J Ford J. Multi-modal deep learning from imaging genomic data for schizophrenia classification. Front Psychiatry. (2024) 15:1384842. doi: 10.3389/fpsyt.2024.1384842, PMID: 39006822 PMC11239396

[43] Zhu X Du X Kerich M Lohoff FW Momenan R. Random forest based classification of alcohol dependence patients and healthy controls using resting state MRI. Neurosci Lett. (2018) 676:27–33. doi: 10.1016/j.neulet.2018.04.007, PMID: 29626649 PMC5960433

[44] Vergara VM Espinoza FA Calhoun VD. Identifying alcohol use disorder with resting state functional Magnetic Resonance Imaging data: a comparison among machine learning classifiers. Front Psychol. (2022) 13:867067. doi: 10.3389/fpsyg.2022.867067, PMID: 35756267 PMC9226579

[45] Kamarajan C Ardekani BA Pandey AK Kinreich S Pandey G Chorlian DB. Random forest classification of alcohol use disorder using fMRI functional connectivity, neuropsychological functioning, and impulsivity measures. Brain Sci. (2020) 10:115. doi: 10.3390/brainsci10020115, PMID: 32093319 PMC7071377

[46] Guggenmos M Schmack K Veer IM Lett T Sekutowicz M Sebold M. A multimodal neuroimaging classifier for alcohol dependence. Sci Rep. (2020) 10:298. doi: 10.1038/s41598-019-56923-9, PMID: 31941972 PMC6962344

[47] Fede SJ Grodin EN Dean SF Diazgranados N Momenan R. Resting state connectivity best predicts alcohol use severity in moderate to heavy alcohol users. Neuroimage: Clin. (2019) 22:101782. doi: 10.1016/j.nicl.2019.101782, PMID: 30921611 PMC6438989

[48] Park J-H Shin Y-B Jung D Hur J-W Pack SP Lee H-J. Machine learning prediction of anxiety symptoms in social anxiety disorder: utilizing multimodal data from virtual reality sessions. Front Psychiatry. (2025) 15:1504190. doi: 10.3389/fpsyt.2024.1504190, PMID: 39896993 PMC11784525

[49] Rapisarda F Lanovaz MJ Guay S Geoffrion S. Machine learning models to predict posttraumatic stress injuries in a sample of firefighters: A proof of concept. Int J Ment Health. (2025), 1–21. doi: 10.1080/00207411.2025.2486084

[50] Shinde SS Ghotkar AS. Mental stress detection with the multimodal data using ensemble optimization enabled explainable convolutional neural network. Biomed Mater Devices. (2025), 1–23.

[51] Chung M Won JB Kim G Kim Y Ozbulak U. (2024). Evaluating visual explanations of attention maps for transformer-based medical imaging, in: International Conference on Medical Image Computing and Computer-Assisted Intervention. 15011:110–20. Springer.

[52] Fritz M Klawonn AM Zahr NM. Neuroimaging in alcohol use disorder: From mouse to man. J Neurosci Res. (2022) 100:1140–58. doi: 10.1002/jnr.24423, PMID: 31006907 PMC6810809

[53] Dang Y Huang K Huo J Yan Y Huang S Liu D. Explainable and interpretable multimodal large language models: A comprehensive survey. (2024). arXiv preprint arXiv:2412.02104.

[54] Wu J. Anomaly detection in medical via multimodal foundation models. Front Bioengineering Biotechnol. (2025) 13:1644697. doi: 10.3389/fbioe.2025.1644697, PMID: 40873433 PMC12378924

[55] Fama R Le Berre A-P Sassoon SA Zahr NM Pohl KM Pfefferbaum A. Relations between cognitive and motor deficits and regional brain volumes in individuals with alcoholism. Brain Structure Funct. (2019) 224:2087–101. doi: 10.1007/s00429-019-01894-w, PMID: 31161472 PMC7082221

[56] Tulay EE Metin B Tarhan N Arikan MK. Multimodal neuroimaging: basic concepts and classification of neuropsychiatric diseases. Clin EEG Neurosci. (2019) 50:20–33. doi: 10.1177/1550059418782093, PMID: 29925268

[57] Stahlschmidt SR Ulfenborg B Synnergren J. Multimodal deep learning for biomedical data fusion: a review. Briefings Bioinf. (2022) 23:bbab569. doi: 10.1093/bib/bbab569, PMID: 35089332 PMC8921642

[58] Calhoun VD Sui J. Multimodal fusion of brain imaging data: a key to finding the missing link (s) in complex mental illness. Biol Psychiatry: Cogn Neurosci Neuroimaging. (2016) 1:230–44. doi: 10.1016/j.bpsc.2015.12.005, PMID: 27347565 PMC4917230

[59] Zhang Y-D Dong Z Wang S-H Yu X Yao X Zhou Q. Advances in multimodal data fusion in neuroimaging: Overview, challenges, and novel orientation. Inf Fusion. (2020) 64:149–87. doi: 10.1016/j.inffus.2020.07.006, PMID: 32834795 PMC7366126

[60] Baltrušaitis T Ahuja C Morency L-P. Multimodal machine learning: A survey and taxonomy. IEEE Trans Pattern Anal Mach Intell. (2018) 41:423–43. doi: 10.1109/TPAMI.2018.2798607, PMID: 29994351

[61] Pu Q Xi Z Yin S Zhao Z Zhao L. Advantages of transformer and its application for medical image segmentation: a survey. Biomed Eng Online. (2024) 23:14. doi: 10.1186/s12938-024-01212-4, PMID: 38310297 PMC10838005

[62] Azad R Kazerouni A Heidari M Aghdam EK Molaei A Jia Y. Advances in medical image analysis with vision transformers: a comprehensive review. Med Image Anal. (2024) 91:103000. doi: 10.1016/j.media.2023.103000, PMID: 37883822

[63] Salvador R Radua J Canales-Rodríguez EJ Solanes A Sarró S Goikolea JM. Evaluation of machine learning algorithms and structural features for optimal MRI-based diagnostic prediction in psychosis. PloS One. (2017) 12:e0175683. doi: 10.1371/journal.pone.0175683, PMID: 28426817 PMC5398548

[64] Sui J Adali T Yu Q Chen J Calhoun VD. A review of multivariate methods for multimodal fusion of brain imaging data. J Neurosci Methods. (2012) 204:68–81. doi: 10.1016/j.jneumeth.2011.10.031, PMID: 22108139 PMC3690333

[65] Koh D Aw T-C. Surveillance in occupational health. Occup Environ Med. (2003) 60:705–10. doi: 10.1136/oem.60.9.705, PMID: 12937199 PMC1740637

[66] Littlejohns TJ Holliday J Gibson LM Garratt S Oesingmann N Alfaro-Almagro F. The UK Biobank imaging enhancement of 100,000 participants: rationale, data collection, management and future directions. Nat Commun. (2020) 11:2624. doi: 10.1038/s41467-020-15948-9, PMID: 32457287 PMC7250878

[67] Grant BF Goldstein RB Saha TD Chou SP Jung J Zhang H. Epidemiology of DSM-5 alcohol use disorder: results from the National Epidemiologic Survey on Alcohol and Related Conditions III. JAMA Psychiatry. (2015) 72:757–66. doi: 10.1001/jamapsychiatry.2015.0584, PMID: 26039070 PMC5240584

[68] Sacks JJ Gonzales KR Bouchery EE Tomedi LE Brewer RD. 2010 national and state costs of excessive alcohol consumption. Am J Prev Med. (2015) 49:e73–9. doi: 10.1016/j.amepre.2015.05.031, PMID: 26477807

