# Supplementary material for: Predicting alcohol use disorder risk in firefighters using a multimodal deep learning model: a cross-sectional study
Source: Front Psychiatry. 2025 Nov 3;16:1643552. doi: 10.3389/fpsyt.2025.1643552 (PMC12620616; doi:10.3389/fpsyt.2025.1643552)
Supplement: Supplementary file 1 [file SupplementaryFile1.docx]

Supplementary Material

# Supplementary Figures


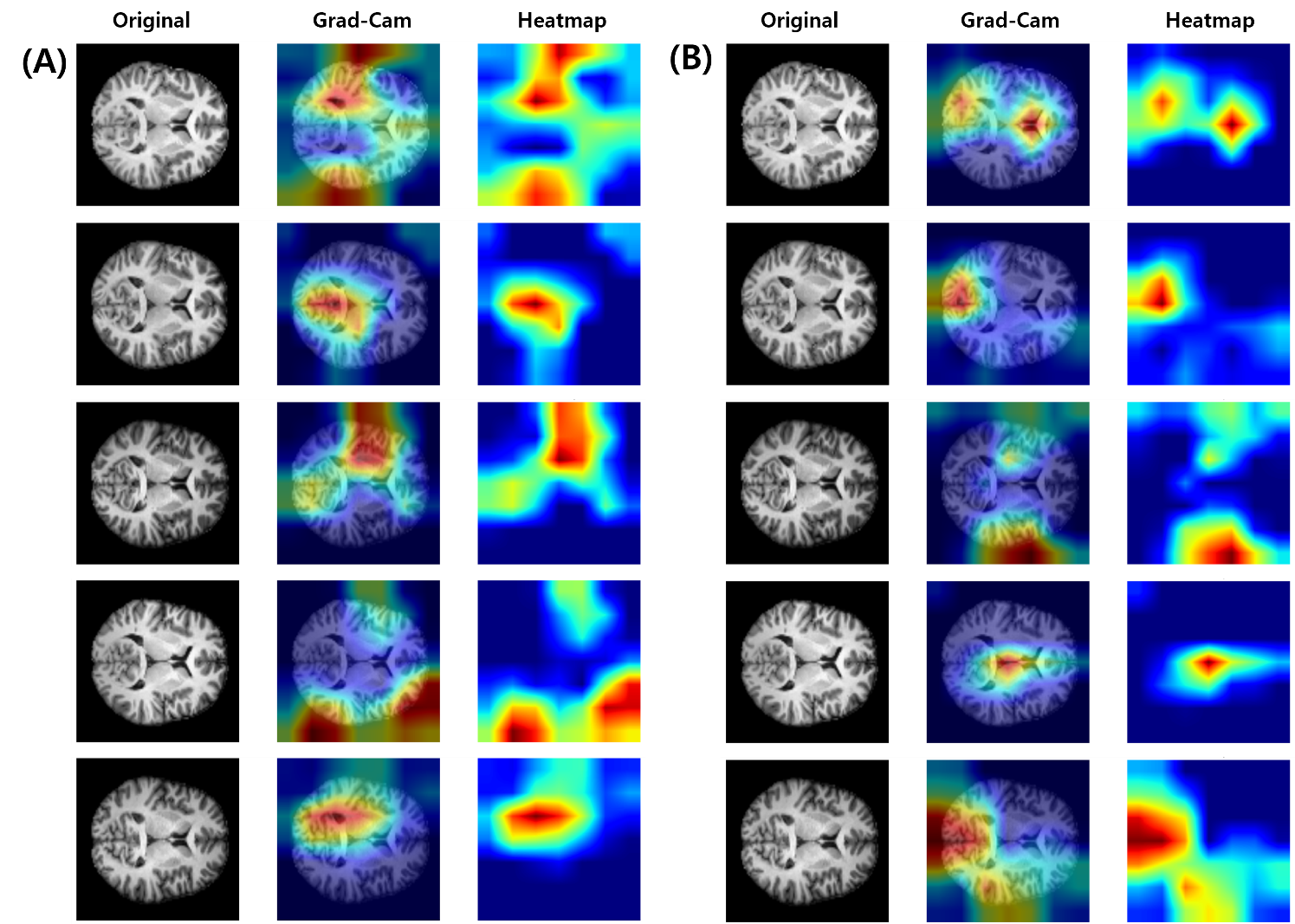


Supplementary Figure 1. Gradient-weighted Class Activation Mapping (Grad-CAM) visualizations of neuroimaging-only models demonstrating stochastic activation patterns.

Representative Grad-CAM visualizations from randomly selected participants showing feature extraction patterns in axial T1-weighted MRI slices. (A) ResNet-50 architecture activation maps. (B) EfficientNet-B0 architecture activation maps. For each panel, columns display (left to right): original preprocessed MRI slice, Grad-CAM overlay on the original image, and isolated heatmap visualization. Rows represent different participants and slice levels. Activation intensities are color-coded from blue (low activation) to red (high activation). Both architectures exhibit heterogeneous, spatially inconsistent activation patterns without systematic concentration in neuroanatomically relevant regions associated with alcohol-related alterations. The stochastic distribution of high-intensity regions across cortical and subcortical territories, lacking anatomical coherence between participants, provides visual evidence for the limited discriminative capacity of structural MRI-only approaches (ResNet-50 AUROC: 57.73%; EfficientNet-B0 AUROC: 56.54%). These visualization patterns corroborate the necessity of multimodal integration to achieve robust alcohol use disorder risk classification in the firefighter cohort.


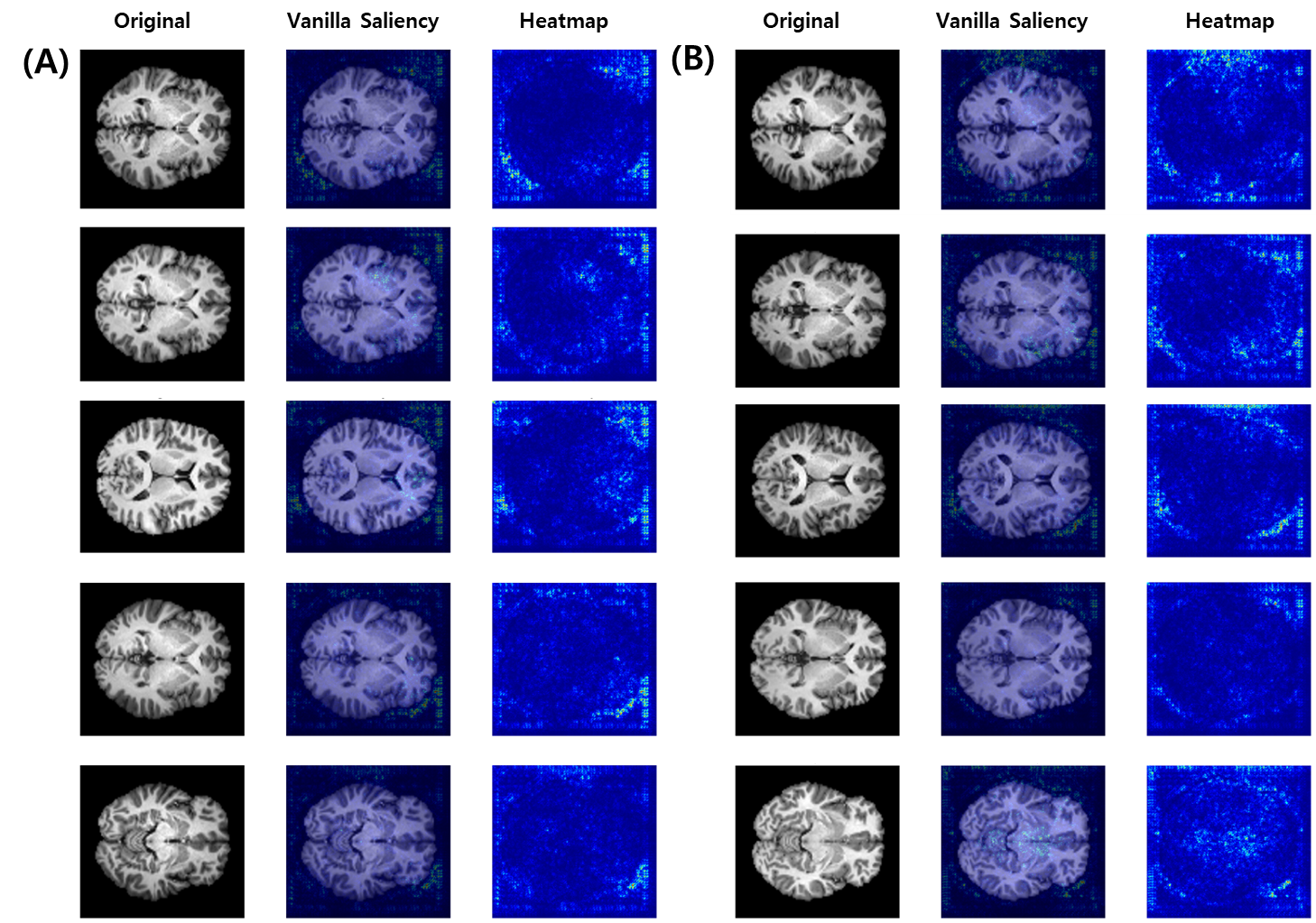


Supplementary Figure 2. Vanilla Saliency visualizations of neuroimaging-only models demonstrating sparse gradient distributions without anatomical specificity.

Vanilla Saliency analysis revealing instantaneous gradient-based feature importance in axial T1-weighted MRI slices using the same models as in Supplementary Figure 1. (A) ResNet-50 architecture. (B) EfficientNet-B0 architecture. For each panel, columns display: original preprocessed MRI slice, saliency overlay, and isolated heatmap. The Vanilla Saliency method computes the gradient of the model's output with respect to the input pixels, providing a first-order approximation of pixel-level importance. Color intensity in the blue spectrum indicates gradient magnitude, with brighter regions representing pixels that most directly influence the model's predictions through single-step backpropagation. The resulting visualizations exhibit characteristic salt-and-pepper noise patterns with sporadic high-gradient pixels scattered throughout the image, reflecting the method's sensitivity to local variations and its tendency to highlight sharp edges and texture boundaries rather than coherent anatomical structures. This granular, pixel-wise attribution pattern demonstrates how the models respond to fine-grained intensity variations without capturing broader morphological patterns relevant to alcohol-related brain changes. Consistent with findings from Grad-CAM visualization (Supplementary Figure 1), these gradient-based patterns lack neuroanatomical coherence, reinforcing the necessity of multimodal integration for robust alcohol use disorder risk classification in the firefighter cohort.


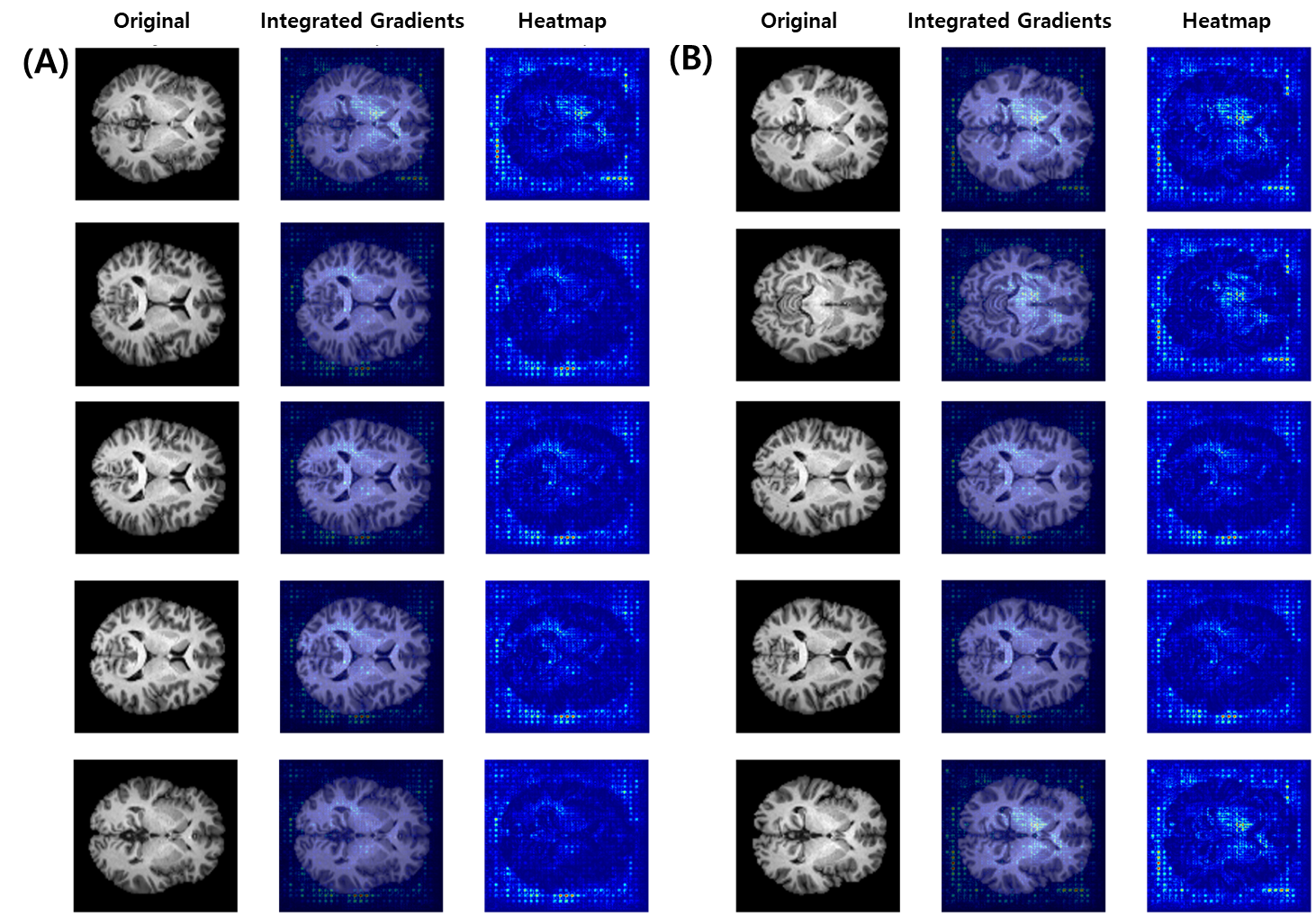


Supplementary Figure 3. Integrated Gradients visualizations of neuroimaging-only models demonstrating punctate attribution patterns lacking neuroanatomical coherence.

Integrated Gradients analysis demonstrating path-integrated attribution accumulation in axial T1-weighted MRI slices using the same models as in Supplementary Figure 1. (A) ResNet-50 architecture. (B) EfficientNet-B0 architecture. For each panel, columns display: original preprocessed MRI slice, integrated gradients overlay, and isolated heatmap. Unlike single-gradient methods, Integrated Gradients computes attributions by accumulating gradients along the straight-line path from a baseline (zero-intensity image) to the actual input, satisfying the axioms of sensitivity and implementation invariance. The visualization employs a blue-to-white gradient scale where dotted white patterns indicate cumulative feature importance. The distinctive punctate, grid-like patterns observed result from the integration process capturing both the presence and absence of signal, creating a more structured attribution map than instantaneous gradient methods. These regular, lattice-like patterns suggest the models learn to detect periodic image features and boundary transitions rather than continuous anatomical regions, with the integration process revealing how features build up from the baseline to form the final prediction. Similar to the spatially inconsistent activation patterns observed with Grad-CAM (Supplementary Figure 1), this alternative visualization approach confirms that structural MRI alone cannot capture the complex neurobiological signatures of alcohol-related changes, necessitating the incorporation of functional neuroimaging and clinical biomarkers.


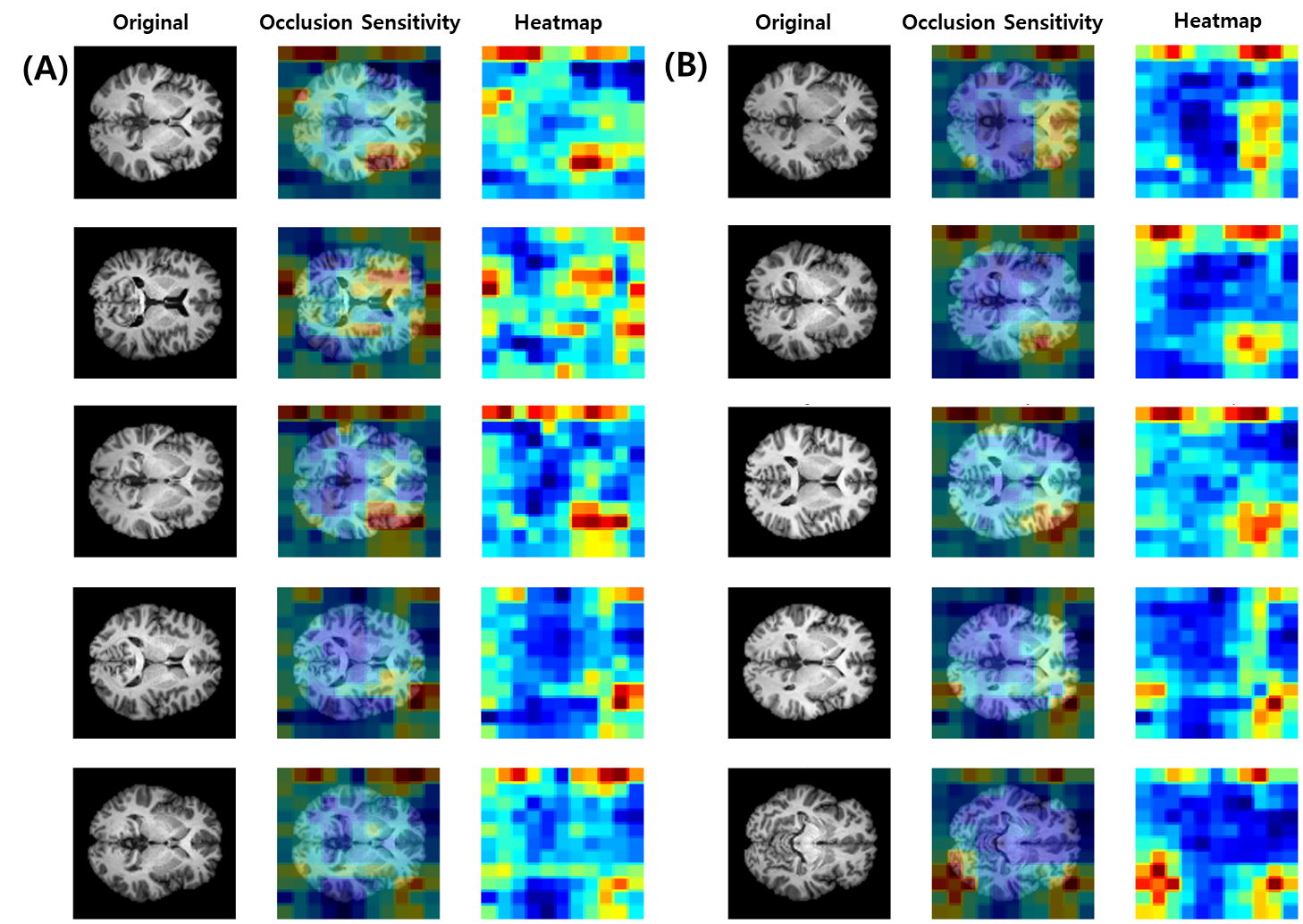


Supplementary Figure 4. Occlusion Sensitivity visualizations of neuroimaging-only models demonstrating heterogeneous regional dependencies without structural consistency.

Occlusion Sensitivity analysis mapping regional prediction dependencies through systematic masking in axial T1-weighted MRI slices using the same models as in Supplementary Figure 1. (A) ResNet-50 architecture. (B) EfficientNet-B0 architecture. For each panel, columns display: original preprocessed MRI slice, occlusion sensitivity overlay, and isolated heatmap. This perturbation-based approach systematically occludes rectangular patches (sliding window) across the input image and measures the resulting change in prediction confidence, directly quantifying each region's contribution to the model output. The multi-color heatmap uses a spectrum from blue (minimal impact when occluded) through cyan and green to yellow and red (maximal impact when occluded), providing a coarse-grained spatial importance map. The characteristic block-like patterns with sharp boundaries between sensitivity levels reflect the sliding window methodology, where each position represents the averaged importance of the occluded patch. Notable regions are the irregular, non-contiguous high-sensitivity regions (red-orange patches) that appear randomly distributed rather than concentrated in neuroanatomically meaningful areas. This occlusion-based analysis provides a complementary perspective to gradient-based methods by directly measuring prediction robustness to regional information loss, revealing that the models lack consistent spatial dependencies that would indicate learning of structural biomarkers. Together with the heterogeneous patterns observed across all visualization methods (Supplementary Figures 1-3), these findings provide converging evidence that multimodal data integration is essential for achieving clinically meaningful alcohol use disorder risk assessment in occupational health screening contexts.


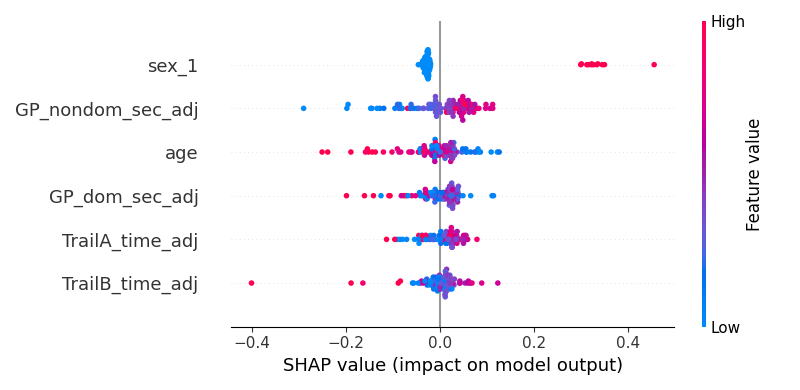


Supplementary Figure 5. SHapley Additive exPlanations (SHAP) feature importance analysis of clinical variables in the multimodal deep learning framework.

SHAP summary plot illustrating the distribution and impact of clinical features on alcohol use disorder risk prediction in the multimodal model. The horizontal axis represents SHAP values indicating the magnitude and direction of each feature's contribution to the model output, with positive values increasing predicted risk probability and negative values decreasing it. Features are arranged vertically in descending order of mean absolute SHAP value importance. Each point represents an individual participant, with color coding from blue (low feature value) to pink/red (high feature value). Sex (sex_1) demonstrates the highest feature importance with predominantly positive SHAP values, indicating male sex as a primary risk factor. Non-dominant hand Grooved Pegboard performance (GP_nondom_sec_adj) shows substantial bidirectional effects, suggesting motor coordination impairments contribute variably to risk assessment. Age exhibits balanced positive and negative contributions centered near zero. Dominant hand Grooved Pegboard (GP_dom_sec_adj) and Trail Making Test Part A (TrailA_time_adj) display moderate bidirectional importance. Trail Making Test Part B (TrailB_time_adj) shows the most concentrated distribution around zero, indicating minimal direct contribution within the multimodal context. These quantitative feature attributions provide mechanistic insights into the relative importance of clinical indicators complementing structural neuroimaging data in the integrated predictive framework.
